# TMEM97 regulates cholesterol biosynthesis and mitochondrial metabolism in gastric carcinoma

**DOI:** 10.1016/j.isci.2026.116773

**Published:** 2026-07-14

**Authors:** Saniye Koç Ada, Yahya Yozbatıran, Bengü Yaren Beyaz, Ceren Sarı, Elif Gelenli Dolanbay, Halil İbrahim Saygı, Ceren Sümer, Cyrille Mesue Njume, Ali Çakmak, Fatma Zehra Sarı, Şeyma Çimen, Mertkaya Aras, Andaç Kılıçkap, Burcu Yücel

**Affiliations:** 1 Science and Advanced Technologies Research Center (BILTAM), Istanbul Medeniyet University, Istanbul 34700, Türkiye; 2 Department of Medical Biochemistry, Faculty of Medicine, Istanbul Medeniyet University, Istanbul 34700, Türkiye; 3 Department of Medical Biology, Institute of Graduate Programs, Istanbul Medeniyet University, Istanbul 34700, Türkiye; 4 Türkiye Cancer Institute, Health Institutes of Turkiye (TUSEB), Istanbul 34718, Türkiye; 5 Department of Histology and Embryology, Faculty of Medicine, Istanbul Medeniyet University, Istanbul 34700, Türkiye; 6 Department of Computer Engineering, Ayazaga Campus, Istanbul Technical University, Istanbul 34469, Türkiye; 7 Dr. Orhan Öcalgiray Molecular Biology-Biotechnology and Genetics Research Center (MOBGAM), Istanbul 34469, Türkiye; 8 Türkiye Health Data Research and Artificial Intelligence Applications Institute, Health Institutes of Türkiye (TUSEB), Istanbul 34718, Türkiye; 9 Regenerative and Restorative Medicine Research Center (REMER), Research Institute for Health Sciences and Technologies (SABITA), Istanbul Medipol University, Istanbul 34810, Türkiye; 10 Department of Medical Biology, Faculty of Medicine, Istanbul Medeniyet University, Istanbul 34700, Türkiye

**Keywords:** TMEM97, cholesterol biosynthesis, oxidative phosphorylation, CARC/CRAC, metabolic reprogramming, mitochondrial bioenergetics

## Abstract

Cancer cells regulate cholesterol levels to sustain proliferation and metabolic adaptation. Here, we show that TMEM97 coordinates cholesterol sensing with oxidative phosphorylation in gastric cancer. Loss of TMEM97 reduced *in vitro* proliferation and impaired xenograft tumor growth. TMEM97 deficiency disrupted cholesterol homeostasis, causing accumulation of the post-lanosterol intermediate follicular fluid meiosis-activating sterol (FF-MAS) under both normal and lipoprotein-deficient serum conditions. Metabolomic and transcriptomic analyses further revealed altered tricarboxylic acid (TCA) cycle activity under lipoprotein-deficient conditions. Structural analysis identified a conserved cholesterol recognition amino acid consensus (CARC) motif in TMEM97, and its deletion reduced cholesterol binding. Collectively, TMEM97 regulates sterol regulatory element binding protein (SREBP)-associated lipid programs, post-lanosterol cholesterol biosynthesis, and mitochondrial bioenergetics. These findings identify TMEM97 as a regulator of cholesterol-dependent metabolic adaptation in gastric cancer and support TMEM97-associated cholesterol regulation as a potential therapeutic vulnerability.

## Highlights

• TMEM97 deficiency disrupts cholesterol synthesis, causing FF-MAS accumulation
• TMEM97 loss activates SREBP feedback, altering lipid metabolic homeostasis
• TMEM97 ablation impairs oxphos and perturbs cellular bioenergetics
• TMEM97 acts as a cholesterol sensor linking lipids to mitochondrial respiration

## Graphical abstract

**Figure:**
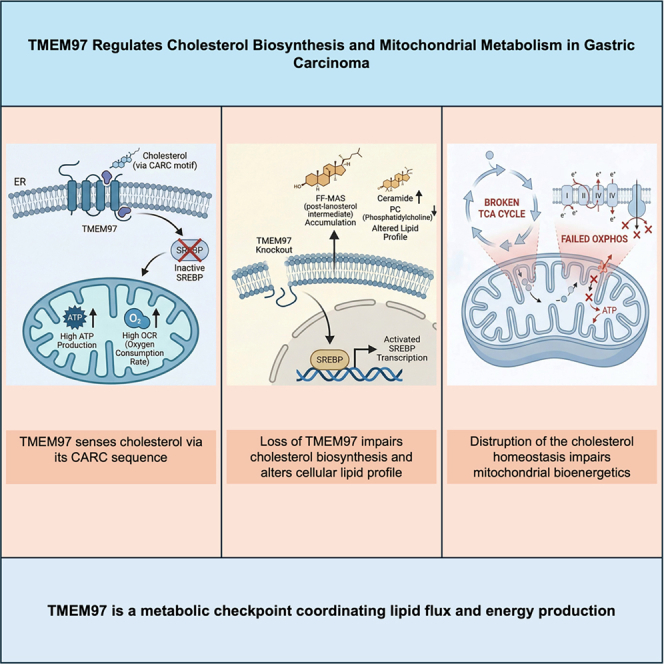

## Teaser

Human metabolism; Cell biology; Cancer

## Introduction

Gastric carcinoma is the fourth leading cause of death from cancer worldwide.^1^ Such as many cancer types, the etiology of gastric carcinoma is multifactorial. Despite recent advances in targeted therapies against actionable mutations, overall prognosis remains unfavorable.^2^ Investigating the interaction between metabolic regulation and carcinogenesis has become an attraction to develop novel therapeutics.^3^ Signaling pathways that drive cell proliferation also regulate the metabolic activity of the cell. Cancer cell interactions with their environment involve multiple-layered crosstalk and result in the dysregulation of canonical signaling pathways.^4^

Cholesterol metabolism is one of the key pathways that support cell growth and proliferation.^5^ In proliferating cells, both endogenous cholesterol synthesis and the uptake of exogenous lipids are necessary to produce new membranes and sustain active cell signaling systems.^6^ Energy-intensive cholesterol biosynthesis is tightly regulated by an efficient cholesterol-sensing mechanism that is activated when extracellular cholesterol is limited, or intracellular levels are insufficient.^7^^,^^8^ Cholesterol sensing emerges proximally to the biosynthetic pathway as sterol-sensing proteins and enzymes that catalyze key steps are co-localized in the endoplasmic reticulum (ER) membrane.^9^

TMEM97 also known as meningioma-associated protein, MAC30, or sigma-2 receptor is an integral membrane protein and localized in the ER membrane, plasma membrane and endolysosomal compartments in the sterol-depleted environment.^10^ Despite its hydrophobic nature, TMEM97 can bind many different small molecule chemotype ligands making it a promising drug target.^11^ TMEM97 has been implicated in multiple aspects of cholesterol homeostasis.^12^ It forms a complex with progesterone receptor membrane component 1 (PGRMC1) and LDLR for the rapid internalization of LDL.^13^ In addition, *TMEM97* expression decreases in sterol depleted cells upon siRNA-mediated knockdown of sterol regulatory element binding protein 2 (SREBP2).^14^ Furthermore, TMEM97 interacts with NPC1, a cholesterol-binding integral membrane protein in lysosomes, to promote cholesterol efflux from lysosomes thereby increasing intracellular cholesterol levels in cholesterol insufficient conditions.^15^

Despite recent findings, it is still unclear whether TMEM97 directly participates in cholesterol biosynthesis or influences intracellular cholesterol distribution. Here, we aimed to investigate the role of TMEM97 in cholesterol homeostasis and its potential impact on carcinogenesis, specifically elucidating whether it exerts regulatory or direct metabolic effects that drive tumor progression.

## Results

### TMEM97 has oncogenic properties in gastric carcinoma

**Figure 1:**
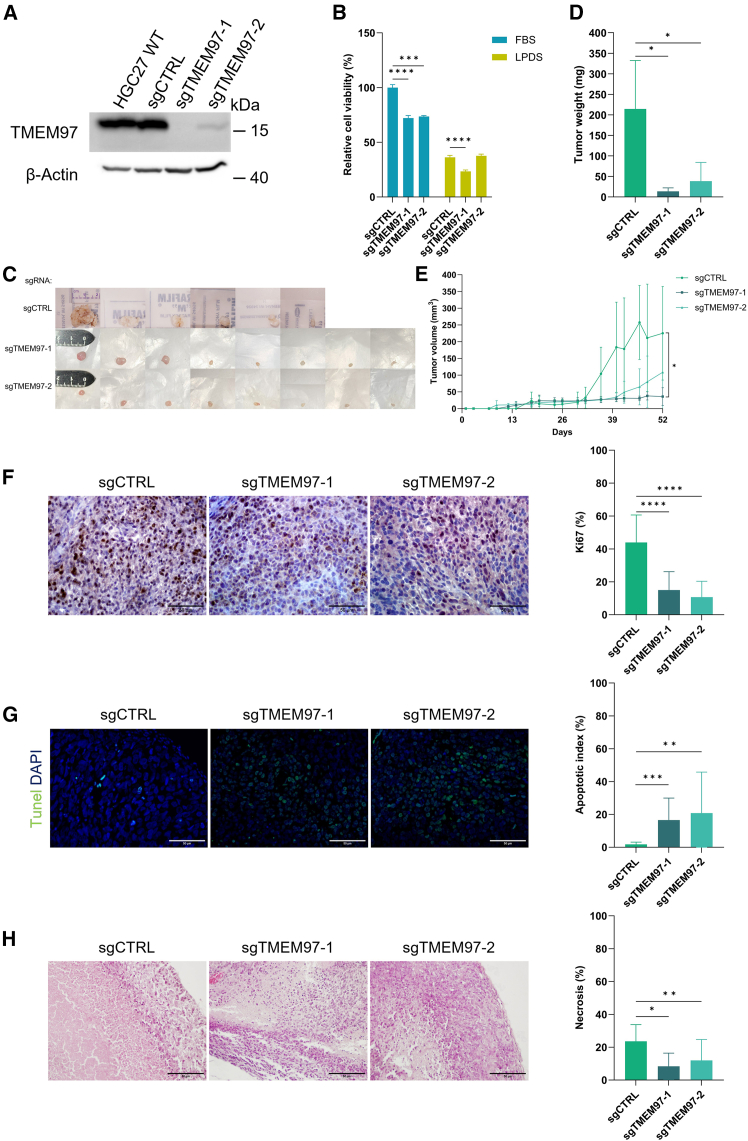
Loss of *TMEM97* reduces tumor growth in gastric carcinoma (A) Representative western blot analysis of whole-cell lysates from HGC27 gastric carcinoma wild-type (WT) and monoclonal cell lines transduced with non-targeting control (sgCTRL) or *TMEM97*-targeting sgRNAs (sgTMEM97-1 and sgTMEM97-2). The uncropped versions of the immunoblots are provided in the Supplemental materials. (B) Cell viability assay (CellTiter-Glo) results of *TMEM97* knockout cells compared to sgCTRL under both FBS and lipoprotein deficient serum (LPDS) conditions (*n* = 5). (C) Representative images of xenograft tumors derived from HGC27 sgCTRL (*n* = 6) and *TMEM97* knockout (*n* = 8 each) cell lines in nude mice. (D) Quantification of tumor volume in xenograft models. (E) Quantification of tumor weight in xenograft models. (F) Immunohistochemical analysis of Ki67 staining in tumor sections. Quantification is based on Ki67-positive cell ratios in randomly selected regions. (G) TUNEL assay of xenograft tumor sections. Nuclei were stained with DAPI (blue) and apoptotic cells with TUNEL (green). (H) Representative H&E images of xenograft tumors from sgCTRL and *TMEM97* knockout groups. Quantification of necrosis (right) shows a significantly higher necrotic area in sgCTRL-derived tumors. Statistical significance was determined using Welch’s *t* test and is indicated as follows: ∗*p* < 0.05, ∗∗*p* < 0.01, ∗∗∗*p* < 0.001, and ∗∗∗∗*p* < 0.0001. Data are presented as mean ± SD. Scale bars represent 50 μm for microscopy images.

*TMEM97* is highly expressed in gastric carcinoma cell lines (supplementary information, Figure S1A), and we validated *TMEM97* mRNA expression in gastric carcinoma cell lines AGS, HGC27, and MKN45 with qRT-PCR (Figure S1B). Analysis of the TCGA gastric carcinoma dataset revealed that tumor samples have significantly higher *TMEM97* expression than normal tissue samples (supplementary information, Figure S1C). To investigate oncogenic properties of TMEM97 in gastric carcinoma, we generated *TMEM97*-knocked out HGC27 cell lines using two distinct sgRNAs, and deletions were validated with Sanger sequencing (supplementary information, Figure S1D). However, following single-cell cloning, one clone showed residual but detectable expression of TMEM97 (Figure 1A), suggesting incomplete knockout or cellular heterogeneity. Despite this difference, both *TMEM97* knockout cells (KOs) were less proliferative compared to the control *in vitro* (Figure 1B). To reveal the tumorigenic potential of TMEM97, xenograft models were established by injecting cells into immunodeficient nude mice (Figure 1C). *TMEM97-deficient* tumor samples had significantly reduced volume and weight, compared to the control group (Figures 1D and 1E). Furthermore, immunohistochemical analysis of Ki67, a proliferation index, showed lower staining in KO tumors, indicating decreased proliferative activity (Figure 1F). Consistently, TUNEL assay revealed increased cell death in KO tumors while H&E staining showed fewer necrotic areas compared to the control group (Figures 1G and 1H).

### TMEM97 involved in cholesterol homeostasis via SREBP-dependent mechanisms

**Figure 2:**
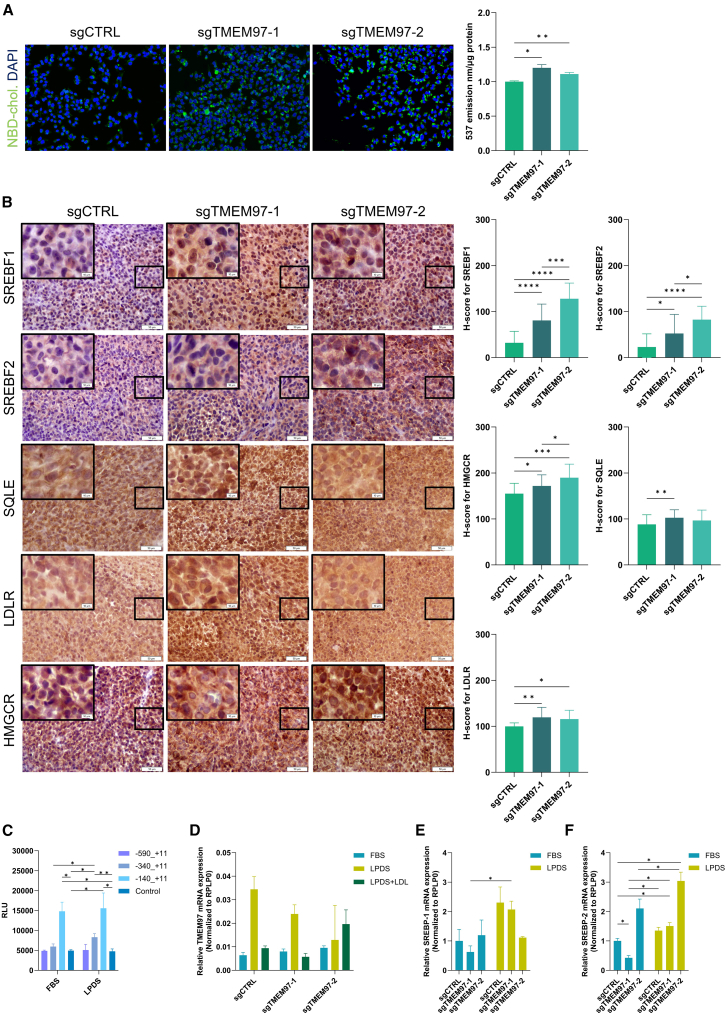
Loss of *TMEM97* decreases cholesterol levels and upregulates the expression of regulatory proteins involved in cholesterol metabolism (A) Representative fluorescence images of nitrobenzoxadiazole (NBD)-cholesterol uptake in sgCTRL and *TMEM97* knockout HGC27 cells cultured in LPDS medium for 24 h. Nuclei are counterstained with DAPI (blue). Quantification of fluorescence intensity (right) normalized to total protein (μg). Images were acquired at 20× magnification. (B) Immunohistochemical staining and H-score quantification of SREBF1, SREBF2, HMGCR, SQLE, and LDLR in sgCTRL and *TMEM97* KO xenograft tumors (*n* = 5 randomly selected areas). Black boxes indicate the areas selected for higher magnification shown in the corresponding top-left insets. Scale bars represent 50 μm for main images and 10 μm for insets. The corresponding graphs displaying the H-scores of the proteins are provided alongside the staining panels. (C) Luciferase reporter assay revealed increased promoter activity driven by SREBP in *TMEM97* knockout cells under either FBS or LPDS conditions. (D) *TMEM97* mRNA expression was measured in sgCTRL and *TMEM97* knockout HGC27 cells cultured under either FBS or LPDS conditions. (E and F) RT-qPCR analysis of *SREBP1* and *SREBP2* mRNA expression under both FBS and LPDS conditions. Statistical significance was determined using Welch’s *t* test and is indicated as follows: ∗*p* < 0.05, ∗∗*p* < 0.01, ∗∗∗*p* < 0.001, and ∗∗∗∗*p* < 0.0001. Data are presented as mean ± SD.

To exploit the function of TMEM97 in cholesterol homeostasis, we first examined how *TMEM97* KO cells respond to lipoprotein deficient serum (LPDS) conditions. Notably, *TMEM97* KO cells displayed impaired proliferation in LPDS medium, compared to parental cells (Figure 1B). Loss of *TMEM97* resulted in an upregulation of *SQLE* and *HMGCR*, two key rate-limiting enzymes in cholesterol biosynthesis, along with elevated *SREBP1* and *SREBP2* expression levels (Figures 2E and 2F), which suggested an activation of a compensatory mechanism in normal medium.

*TMEM97* KO cells exhibited elevated cholesterol uptake as detected by nitrobenzoxadiazole (NBD)-cholesterol assay and Dil-LDL assay (Figure 2A and supplementary information; Figure S2A) in both normal and LPDS medium. Consistent with the enhanced lipid uptake, increased LDLR expression was detected in *TMEM97* KO cells both *in vitro* in normal medium and *in vivo* in a xenograft model Figures 2B and S2B). Moreover, knockout cells elevated their *LDLR* expression in the LPDS medium, and this effect was diminished by the addition of LDL into the medium, indicating normal lipid uptake (Figure 2F). High cytoplasmic LDLR staining was detected in KO cells compared to controls, implying increased endocytosis of LDLR, as *PCSK9* gene expression was also found elevated (supplemental information, Figures S2D, S2B, and S2C).

To determine whether *TMEM97* expression is regulated by cholesterol availability, we evaluated *TMEM97* promoter activity in the LPDS condition. Luciferase reporter assays showed a significant increase in promoter activity of TMEM97 in LPDS-treated cells compared to normal serum conditions (Figure 2C). In line with this, *TMEM97* mRNA expression was also elevated in LPDS medium, which displays a transcriptional induction in parallel with *SREBP* activation (Figures 2D–2F). Overall, these findings indicate that TMEM97 is involved in a feedback loop in the SREBP2 regulatory network in cholesterol homeostasis.

### Loss of TMEM97 alters the levels of post-lanosterol intermediates

**Figure 3:**
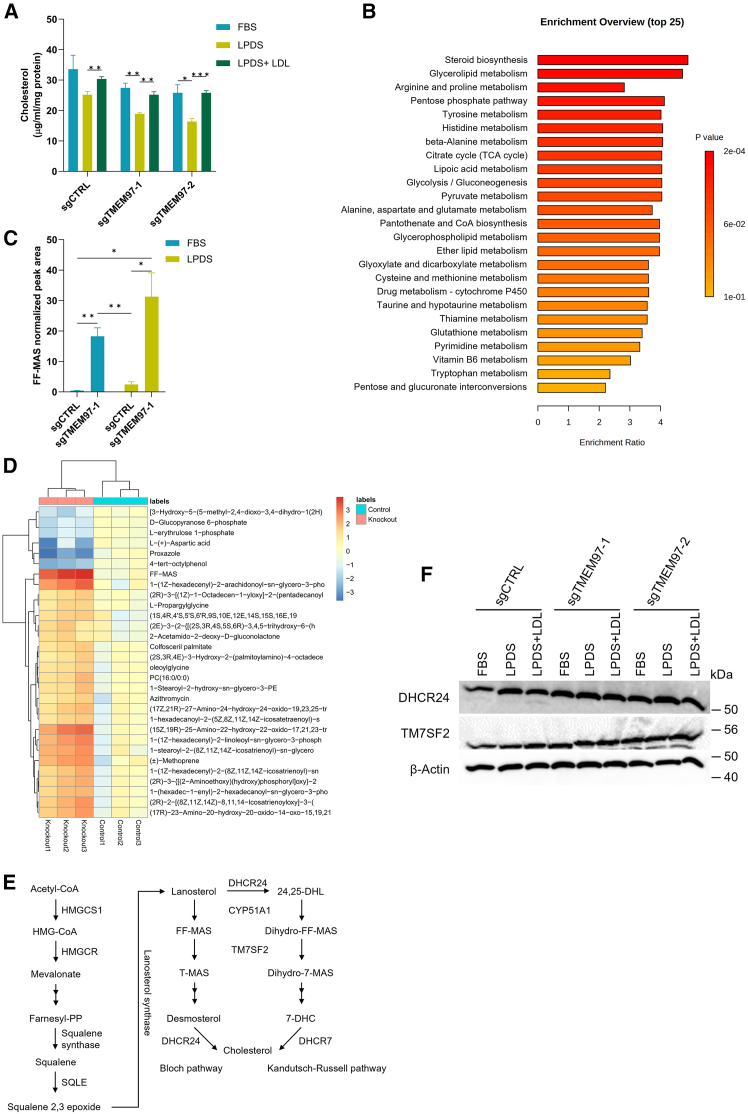
Ablation of *TMEM97* impairs cholesterol biosynthesis and leads to FF-MAS accumulation (A) Total cellular cholesterol was quantified by Amplex Red Cholesterol assay in sgCTRL and *TMEM97* knockout HGC27 cells cultured in 10% FBS, LPDS, or LPDS + LDL (50 μg/mL) (*n* = 3). (B) KEGG pathway enrichment analysis of the top 25 detected metabolites in *TMEM97* knockout cells cultured under LPDS conditions. Enrichment was performed using MetaboAnalyst. (C) Bar graph shows the normalized peak area of FF-MAS in sgCTRL and sgTMEM97-1 HGC27 cells cultured in either FBS or LPDS medium. (D) Heatmap based on the top 30 differentially abundant metabolites. (E) Schematic representation of lanosterol-derived sterol metabolism pathways. (F) Protein expression of TM7SF2 and DHCR24 in *TMEM97* knockout cells under FBS and LPDS culture conditions. The uncropped versions of the immunoblots are provided in the Supplemental materials. Statistical significance was determined using Welch’s *t* test and is indicated as follows: ∗*p* < 0.05 and ∗∗∗∗*p* < 0.0001. Data are presented as mean ± SD.

Next, total cholesterol level was measured in KO cells cultured in LPDS medium. Results showed that the abolishment of *TMEM97* further reduced cholesterol levels in HGC27 cells, and this effect was diminished by supplementing LDL into the medium, indicating impaired cholesterol biosynthesis rather than lipid uptake (Figure 3A). To further investigate impaired cholesterol biosynthesis, untargeted metabolomics was applied. Untargeted metabolomics analysis highlighted steroid biosynthesis as one of the top enriched pathways in *TMEM97* knockout (KO) cells cultured in both FBS and LPDS conditions, suggesting altered cholesterol metabolism (Figure 3B and supplementary information; Figure S3A). Differential metabolite analysis revealed a marked accumulation of follicular fluid meiosis-activating sterol (FF-MAS; 14-demethyl-14-dehydrolanosterol) in *TMEM97* KO cells, which was further elevated in LPDS medium (Figures 3C and 3D and supplementary information; Figure S3B). FF-MAS is converted to testis meiosis-activating sterol (T-MAS) via TM7SF2 in the Bloch pathway or to dihydro-FF-MAS via DHCR24 in the Kandutsch-Russell pathway (Figure 3E). In line with previous findings, DHCR24 protein level is not changed by cholesterol level.^16^ On the contrary, TM7SF2 protein expression has been reported to be sterol-sensitive, although we did not observe significant change in LPDS-containing medium.^17^ TM7SF2 and DHCR24 were expressed at comparable levels in KO and control cells (Figure 3F). Additionally, a co-immunoprecipitation assay using GFP-tagged TMEM97 showed no direct interaction between TMEM97 and TM7SF2 or DHCR24 under the conditions tested (Figure 3G).

### TMEM97 deficiency impairs TCA cycle and mitochondrial metabolites

**Figure 4:**
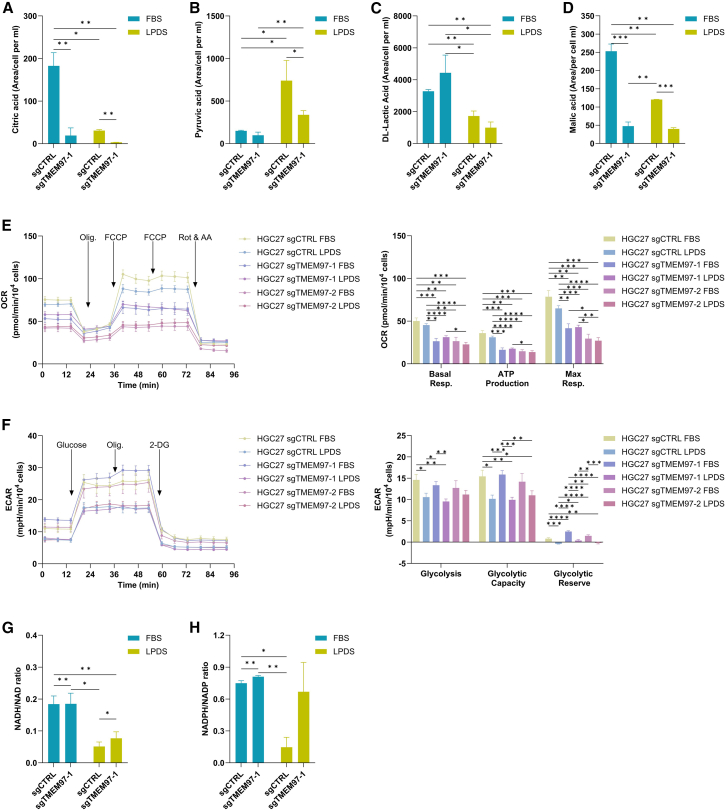
*TMEM97* deficiency impairs mitochondrial metabolism and energy production under lipid-deprived conditions (A–F) Relative abundance of citric acid (A), pyruvic acid (B), DL-lactic acid (C), and malic acid (D) in sgTMEM97-1 and sgCTRL HGC27 cells cultured in FBS or LPDS conditions. (E) Oxygen consumption rate (OCR), including basal respiration, ATP production, and maximal respiratory capacity, measured by Seahorse XF analysis in sgCTRL and TMEM97 knockout cells cultured in FBS or LPDS conditions. (F) Extracellular acidification rate (ECAR), including glycolysis, glycolytic capacity, and glycolytic reserve, measured by Seahorse XF analysis in sgCTRL and *TMEM97* knockout cells cultured in FBS or LPDS. (G and H) Ratio of redox-related cofactors NADH/NAD (G), NADPH/NADP (H) in sgCTRL and *TMEM97* knockout HGC27 cells cultured in FBS or LPDS. Statistical significance was determined using Welch’s *t* test and is indicated as follows: ∗*p* < 0.05, ∗∗*p* < 0.01, ∗∗∗*p* < 0.001, and ∗∗∗∗*p* < 0.0001. Data in graphs are presented as mean ± SD.

Metabolomics analysis highlighted the TCA cycle as one of the significantly altered pathways in metabolite profiling (Figure 3B and supplementary information; Figure S3A). Citric acid level was observed to be reduced in *TMEM97* KO cells both in complete and LPDS-containing media, implying enhanced conversion to supply cytosolic acetyl-CoA for lipid and cholesterol synthesis (Figure 4A). Consistently, pyruvic acid, malic acid, and lactic acid levels were detected at lower levels in KO cells compared to control cells in the LPDS medium (Figures 4B–4D). In addition, palmitic acid levels decreased in lipoprotein-deficient conditions, indicating increased mitochondrial oxidation; however, we did not observe a significant difference between *TMEM97* KO and control cells in either medium (supplementary information, Figure S4A). Furthermore, α-ketoglutarate (α-KG) was detected at elevated levels in KO cells under both conditions, while glutamine levels were not significantly changed between groups (supplementary information, Figures S4B and S4C). In LPDS medium, *TMEM97* KO cells exhibited a higher NADPH/NADP ratio compared to controls, suggesting changes in the NADPH utilization, potentially due to defective post-lanosterol cholesterol biosynthesis (Figures 4G and 4H). Oxygen consumption rate (OCR) measurement confirmed diminished OCR, adenosine triphosphate (ATP) production, and maximal respiratory capacity in KO cells (Figure 4E). In extracellular acidification rate (ECAR) assay, glycolysis rate and glycolytic capacity were unchanged in FBS but decreased in LPDS, suggesting a metabolic transition from TCA to cholesterol biosynthesis (Figure 4F).

### TMEM97 ablation induces transcriptional changes in mitochondrial genes

**Figure 5:**
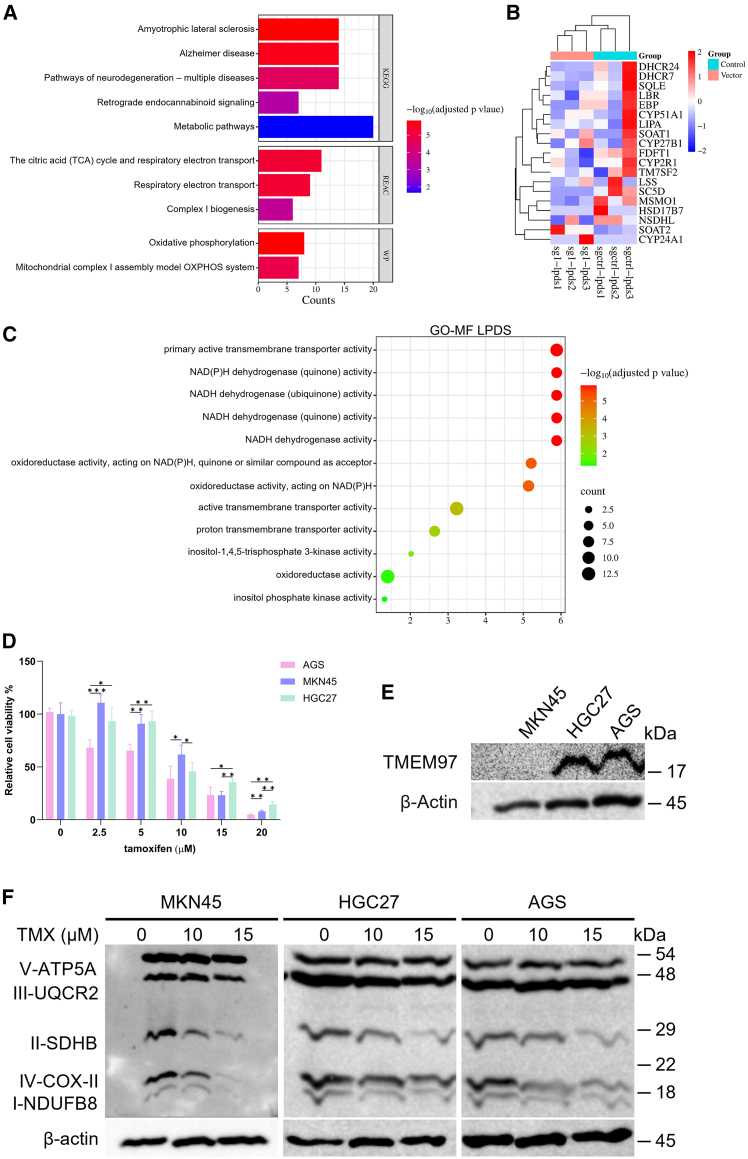
*TMEM97* deficiency induces transcriptional changes in mitochondrial and metabolic pathways under lipid-depleted conditions (A) Pathway enrichment analysis (based on Reactome, KEGG, and WikiPathways) of DEGs identified under LPDS conditions revealed significant enrichment in mitochondrial metabolic processes. Significantly upregulated and downregulated genes are highlighted based on adjusted *p* value <0.05 and |log_2_FC| > 1. (B) Heatmap of genes involved in cholesterol biosynthesis. (C) Validation of mRNA expression levels of the genes in sgCTRL and *TMEM97* KO cells under LPDS conditions with qRT-PCR. (C) Gene Ontology analysis of molecular functions (GO-MF) revealed significant enrichment in redox-associated enzymatic activities. (D) Relative cell viability of AGS, MKN45 and HGC27 cell lines upon tamoxifen treatment (0, 2.5, 5, 10, 15 and 20 μM tamoxifen) (*n* = 3). (E) Western blot analysis of TMEM97 protein expression levels in AGS, MKN45 and HGC27 cell lines, with β-Actin as the loading control. The uncropped versions of the immunoblots are provided in the Supplemental materials. (F) Western blot analysis of OxPHOS protein expression (β-Actin as loading control) in HGC27, AGS and MKN45 cell lines following treatment with increasing doses of tamoxifen (0–20 μM). The uncropped versions of the immunoblots are provided in the Supplemental materials. Statistical significance was determined using Welch’s *t* test and is indicated as follows: ∗*p* < 0.05, ∗∗*p* < 0.01, ∗∗∗*p* < 0.001, and ∗∗∗∗*p* < 0.0001. Data in graphs are presented as mean ± SD.

Next, we evaluated transcriptional changes upon *TMEM97* deletion to determine its broader impact on the metabolic and signaling networks. Bulk RNA-seq analysis was applied to isogenic cell lines cultured in either FBS or LPDS-containing medium. Differential gene expression (DGE) analysis revealed 68 genes were upregulated while 55 genes were downregulated in lipoprotein-deficient medium when the filter was set to a Log2 1-fold change, and pAdj was below 0.05, while 162 genes were detected upregulated. Furthermore, 235 genes were found downregulated in FBS-containing medium. Enrichment analysis revealed the citric acid (TCA) cycle and respiratory electron transport pathway in lipoprotein-deficient medium (Figure 5A). Notably, we did not observe significant changes in gene expression related to cholesterol biosynthesis between control and KO cells in medium with LPDS (Figure 5B and supplementary information; Figure S5D). Gene ontology analysis highlighted enrichment in NAD(P)H dehydrogenase (quinone) activity and NADH dehydrogenase (ubiquione) pathways (Figure 5D). However, in normal medium conditions, TMEM97 ablation induced a transcriptional program to increase protein synthesis (supplementary information, Figures S5A and S5B). Furthermore, GO-BP results indicated that the ablation of *TMEM97* triggers a profound metabolic reprogramming possibly to compensate for impaired cholesterol biosynthesis (supplementary information, Figure S5C). Overall, RNA-seq analysis revealed that *TMEM97* KO cells exhibited a transcriptional response under metabolic stress, presumably to activate master regulatory pathways. Given that TMEM97 has an Emopamil-binding protein (EBP) domain,^18^ we investigated whether pharmacological inhibition of EBP could recapitulate aspects of the *TMEM97* KO phenotype. Gastric cancer cell lines AGS, HGC27, and MKN45 were treated with tamoxifen (TMX) at increasing concentrations (0, 2.5, 5, 10, 15, and 20 μM) (Figure 5D). AGS and HGC27 cell lines displayed relative sensitivity to TMX as compared to MKN45 cells, which lack TMEM97 protein expression (Figure 5E), consistent with previous findings in breast cancer cells.^19^ Notably, TMX treatment led to a dose-dependent reduction in OXPHOS protein levels, indicating impaired mitochondrial function (Figure 5F and supplementary information; Figure S5E). Overall, our data suggest that the inhibition of post-lanosterol biosynthesis disrupts mitochondrial bioenergetics.

### TMEM97 deficiency regulates lipid programming via SREBP1-dependent mechanisms

**Figure 6:**
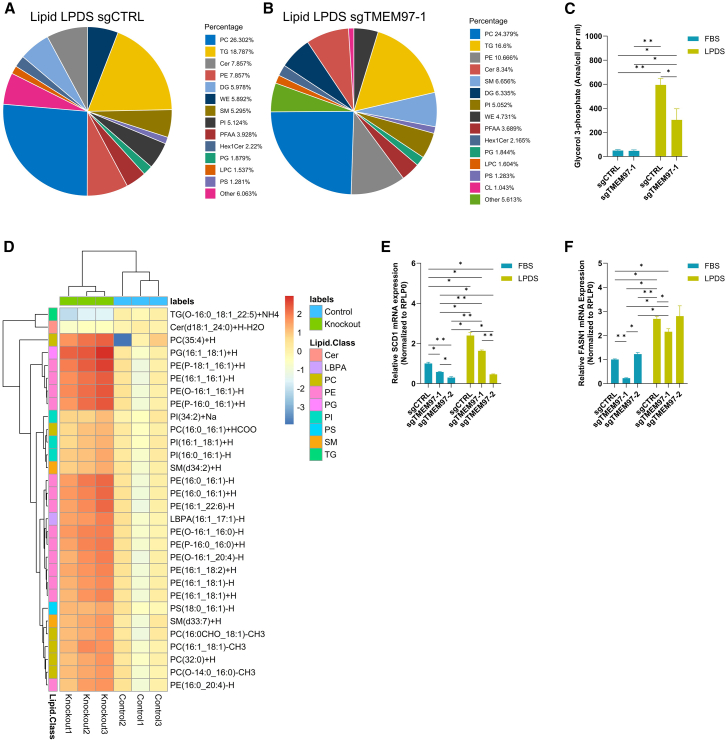
*TMEM97* deficiency alters lipid remodeling under lipoprotein-deprived conditions (A and B) Pie charts display the distribution of lipid classes in sgCTRL (A) and sgTMEM97-1 (B) HGC27 cells cultured under LPDS conditions. (C) Relative abundance of glycerol-3-phosphate (G3P) in sgCTRL and sgTMEM97-1 cells cultured in FBS or LPDS. (D) Heatmap shows the top 30 most differentially expressed lipid species under LPDS conditions in sgCTRL and sgTMEM97-1 cells (E and F) Relative mRNA levels of *FASN1* and *SCD1* were quantified by qRT-PCR in sgCTRL and *TMEM97* knockout cells cultured in FBS and LPDS. Statistical significance was determined using Welch’s *t* test and is indicated as follows: ∗*p* < 0.05, ∗∗*p* < 0.01, and ∗∗∗*p* < 0.001. Data are presented as mean ± SD.

As cholesterol biosynthesis is closely linked to lipid metabolism, untargeted lipidomics was applied to *TMEM97* knockout (KO) cells cultured in either FBS or LPDS (Figures 6A and 6B; Figures S6A and S6B). Elevated glycerol-3-phosphate (G3P) levels were detected in LPDS medium; however, KO cells exhibited lower G3P levels compared to control cells (Figure 6C). Consistently, levels of ceramide, phosphatidylserine, and phosphatidylethanolamine (PE) were significantly increased in KO cells in LPDS-containing medium (Figures 6D and 6E), whereas reduced phosphatidylcholine (PC) and phosphatidylinositol levels were detected in lipoprotein-deficient medium (Figures 6A, 6D, and 6E). Elevated ceramide levels mediate the activation of apoptotic pathways in cancer cells,^20^ which aligns with our observation of increased apoptosis in *TMEM97* KO cells. In addition, the reduced PC/PE ratio affects many cellular processes such as increased SREBP1 activity, ER stress, and cell membrane leakage.^21^ Consistent with impaired TCA cycle, Acylcarnitine (AcCa(16:1)+H) was found higher in *TMEM97* KO cells in FBS medium, while in LPDS medium, its level drastically decreased (Figure 6E), suggesting reduced β-oxidation in lipid depleted conditions. Furthermore, the increase in Triglyceride TG(14:1_16:1_16:1)+Na levels in LPDS medium indicates upregulated fatty acid synthesis (Figure 6E). Collectively, the enhanced expression of *SREBP1*, along with increased *SCD1* and *FASN* mRNA expressions under lipoprotein-deficient conditions, support TMEM97 as a regulator in lipid metabolism via SREBP1-dependent mechanisms (Figures 6F and 6G).

### TMEM97 acts as a cholesterol-sensing protein in the ER membrane

**Figure 7:**
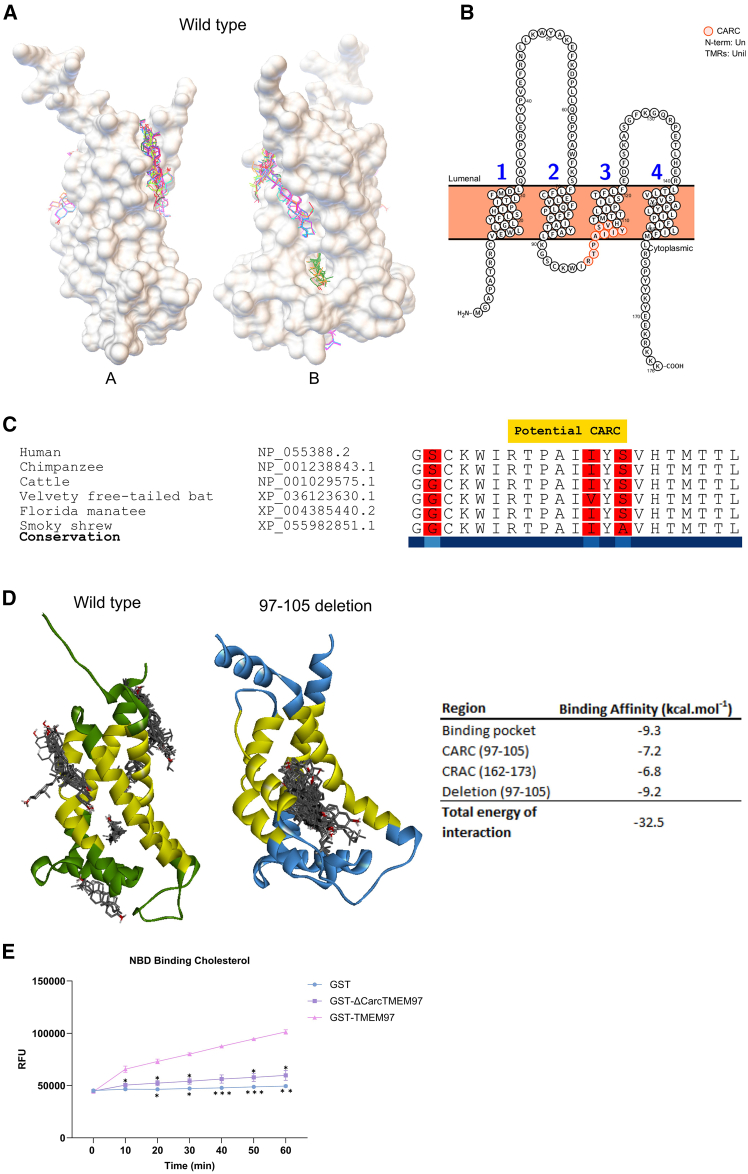
A conserved CARC motif within *TMEM97* contributes to cholesterol binding in the ER membrane (A) Structural docking on an AlphaFold-predicted TMEM97 model identified multiple potential cholesterol interaction sites, including a conserved CARC-containing region. (B) Schematic representation of TMEM97 membrane topology based on AlphaFold-derived predictions. The ER membrane is shown as an orange band with the luminal side above and the cytosolic side below. Four transmembrane helices (TMRs 1–4) are numbered in blue. Residues are shown as circles, with the conserved CARC motif (residues 97–105) in red. (C) Multiple sequence alignment of TMEM97 orthologs from representative vertebrate species, illustrating strict conservation of the CARC motif (residues 97–105). (D) Molecular docking and energy calculations reveal that removal of the conserved CARC sequence impairs cholesterol engagement. Left: Docking of cholesterol (gray sticks) onto AlphaFold-derived TMEM97 models shows altered ligand orientation and loss of the CARC binding pocket in the ΔCARC mutant (blue) compared to wild-type (green). Right: Quantified binding affinities for the mutant CARC site was −9.2 kcal mol^−1^ for the same binding pocket. (E) *In vitro* cholesterol binding assay using GST-tagged wild-type and ΔCARC TMEM97 proteins (1 μM) incubated with 500 nM NBD-cholesterol in PBS; fluorescence emission was recorded every 10 min for 60 min. Statistical significance was determined using Welch’s *t* test and is indicated as follows: ∗*p* < 0.05, ∗∗*p* < 0.01, ∗∗∗*p* < 0.001, and ∗∗∗∗*p* < 0.0001. Data are presented as mean ± SD.

TMEM97 was reported to bind 20(*S*)-hydroxycholesterol, an oxysterol, in cell membranes.^22^ Furthermore, docking analysis against a crystal structure highlighted a binding pocket for cholesterol in the intermembrane section of the protein.^23^ We investigated the potential presence of unique cholesterol recognition amino acid consensus (CARC) and CRAC sequences in TMEM97 (Table S2). Structural docking using an AlphaFold-derived model revealed the same binding pocket in addition to three alternative binding sites, one with a unique CARC sequence (Figure 7A). The motif had a binding affinity of −7.2 kcal/mol with cholesterol. The predicted binding pocket showed the strongest affinity (−9.3 kcal/mol), while the other CRAC motif spanning residues 162–173 had a weaker interaction (−6.8 kcal/mol). Notably, deletion of the 97–105 CARC motif reduced cholesterol binding, with a binding affinity of −9.2 kcal/mol for the same binding pocket. CARC sequences are preferentially found in the luminal leaflet of transmembrane domains and are highly conserved (Figures 7B and 7C). In addition, deletion of this motif (ΔCARC) substantially reduced cholesterol binding *in vitro*, confirming its functional role (Figures 7D and 7E; Figures S7A and S7B). Collectively, these results imply that TMEM97 acts as a cholesterol-sensing protein and modulates intracellular lipid metabolism.

## Discussion

TMEM97 a multi-pass transmembrane protein has emerged with various functions including tumor progression and cholesterol homeostasis.^24^ *TMEM97* previously was identified as an oncogene in colorectal cancer by the induction of GSK-3β/β-catenin pathway^25^ and in breast cancer, through suppressed cell proliferation, colony formation, migration and invasion.^26^ Additionally, in gastric carcinoma cells, downregulation of *TMEM97* expression inhibited proliferation and metastasis ability.^27^ Our results corroborate and extend these observations, where the loss of *TMEM97* reduced tumor growth and increased apoptosis in gastric carcinoma cell line HGC27. These findings pinpointed oncogenic features of *TMEM97* and revealed it as a potential driver of tumorigenesis in gastric carcinoma.

In addition to its oncogenic functions, TMEM97 has been increasingly implicated in cholesterol homeostatis.^13^ *TMEM97* was found among the upregulated genes along with cholesterol biosynthesis in response to progesterone treatment.^28^ TMEM97 interacts with NPC1 on lysosomal membranes to regulate cholesterol trafficking^15^ and joins the LDLR/PGRMC complex for cholesterol uptake.^29^ TMEM97 responds to lipoprotein depletion, and it is a transcriptional target of SREBP2.^30^ Consistent with these findings, in this study, *TMEM97* KO cells displayed enhanced SREBP1/2 expression *in vivo* and even in normal cell culture conditions, indicating a regulatory feedback loop in the SREBP-mediated regulation of cholesterol homeostasis.

Here, we found that *TMEM97* KO cells exhibited increased LDL uptake but reduced intracellular cholesterol levels, implying impaired cholesterol synthesis while having intact exogenous uptake. To further investigate this discrepancy, untargeted metabolomics revealed the accumulation of FF-MAS in the loss of *TMEM97* both in normal and LPDS-containing medium, suggesting a bottleneck at the level of lanosterol conversion. Additionally, in *TMEM97* KO cells, upregulation of *HMGCR* and *SQLE* expression, which are the enzymes that catalyze regulatory steps in cholesterol biosynthesis, could be a result of a compensatory transcriptional response mediated by SREBP activation. Increased SREBP1/2 protein expression in *TMEM97* KO cells also supports this hypothesis. In line with these findings, *TMEM97* KO cells displayed a higher NADPH/NADP ratio in LPDS medium, potentially reflecting an altered utilization of NADPH in cholesterol biosynthesis.^31^

To address whether loss of *TMEM97* impacts metabolic and signaling pathways at the transcriptional level, we performed RNA-seq analysis. Our results showed that along with cholesterol metabolism, the loss of *TMEM97* induced a wide range of metabolic reprogramming. KO cells had reduced levels of TCA cycle intermediates and diminished ATP production, indicating an impairment in oxidative phosphorylation. These findings were aggravated in LPDS medium, suggesting a functional relation between TMEM97, mitochondrial metabolism, and lipid availability. Furthermore, Seahorse Flux analysis confirmed impaired oxidative phosphorylation, while glycolysis was not affected, pointing to altered mitochondrial bioenergetics as cells shift their metabolism toward cholesterol biosynthesis. In addition, the loss of *TMEM97* triggered a wide range of transcriptional remodeling in both FBS and LPDS media, implying that its regulatory function is not limited to adaptive responses to stress conditions but also contributes to basal metabolic homeostasis. In support of a broader role of metabolic regulation of TMEM97, it has been previously shown that its pharmacological activation enhances metabolic activity including increased ATP level, NAD/NADH ratio and elevated glycolytic activity.^32^ In addition to its metabolic function, TMEM97 has been shown to regulate store-operated calcium entry (SOCE) via the STIM1-Orai1 axis, to promote proliferation in triple negative breast cancer cell lines^33^

Given its emerging role in cholesterol metabolism and signaling networks, TMEM97 recently gained attention as a therapeutic target,^34^ and it has been identified as a highly druggable protein.^35^ Recently, it has been shown that cytotoxic ligands of TMEM97 enhance intracellular cholesterol accumulation, triggering ER stress and subsequent cancer cell death.^36^

Reports to identify ligands for TMEM97 showed that it interacts with sterols such as 20(*S*)-hydroxycholesterol, an oxysterol, in cell membranes.^22^ In our study, docking analysis against the AlphaFold structure showed a cholesterol-binding site with the conservative CARC sequence, suggesting TMEM97 as a cholesterol-sensing protein.^37^

To explore the molecular basis underlying the therapeutic potential of TMEM97, we also investigated its structural features and interactions to cholesterol biosynthesis enzymes. TMEM97 has an EBP domain and belongs to the extended EBP (EXPERA) family.^14^ EBP is responsible for the conversion of zymosterol and zymosterol to their corresponding isomers.^38^ However, in line with previous finding,^23^ our data do not support an enzymatic activity of TMEM97 at these steps in cholesterol biosynthesis. Furthermore, co-immunoprecipitation experiments did not show an interaction with enzymes TM7SF2 or DHCR24, suggesting a potential role for TMEM97 in coordinating metabolic responses linked to cholesterol levels. On the other hand, the presence of the EBP domain in the *TMEM97* gene could be exploited to develop therapeutics or to repurpose existing drugs.^39^ For instance, Tamoxifen, an estrogen receptor antagonist used in the treatment of breast cancer, is known to be an EBP inhibitor.^19^

Despite these insights, the lack of enzymatic activity measurements for DHCR24 and TM7SF2 is a limitation that merits consideration. In addition, stable isotope tracing using labeled acetate would also help to support the accumulation of FF-MAS and shift toward cholesterol biosynthesis over the TCA cycle. Furthermore, the question that arises from these findings and needs to be clarified in future studies is to what extent TMEM97’s effects on metabolism are cell/tissue type specific.

In conclusion, our study delineates TMEM97 as a regulator of cholesterol biosynthesis and is linked with mitochondrial metabolism in gastric carcinoma. The abolishment of *TMEM97* alters cholesterol biosynthesis at the post-lanosterol level, induces compensatory activation of SREBP-mediated pathways, and impairs mitochondrial functions. TMEM97 acts as a non-enzymatic regulator and has a potential cholesterol-sensing function through its conserved CARC motif and coordinates metabolic responses to lipid availability. Given its tumorigenic activity, TMEM97 represents a compelling therapeutic target in gastric carcinoma. Future investigations should be employed to further elucidate TMEM97 mechanistic roles and drug development against it.

### Limitations of the study

Although our data support a role for TMEM97 in cholesterol binding and regulation of cholesterol homeostasis, there are several limitations. First, the precise molecular mechanisms linking TMEM97-dependent cholesterol sensing to downstream metabolic reprogramming remain to be fully elucidated. Second, our findings were obtained using gastric cancer cell lines and xenograft models and would benefit from further validation in clinical samples. Finally, future studies will be required to determine how TMEM97-mediated cholesterol regulation contributes to the broader metabolic alterations observed following TMEM97 loss.

## Resource availability

### Lead contact

Requests for further information and resources should be directed to and will be fulfilled by the lead contact, Burcu Yucel (burcu.yucel@medeniyet.edu.tr).

### Materials availability

This study did not generate new unique reagents. Plasmids generated for this study are available from the lead contact.

### Data and code availability

• The RNA-seq raw data reported in this paper have been deposited in the NCBI Gene Expression Omnibus (GEO) and are publicly available as of June 15, 2026. The accession number is listed in the key resources table (GEO: GSE312758). LC-MS/MS raw data will be made available upon requests. All other supporting data are presented as supplementary materials.
• This paper does not report original code.
• Any additional information required to reanalyze the data reported in this paper is available from the lead contact upon request.

## Acknowledgments

We thank Molecular Medicine Research Lab members for their kind help and understanding throughout this project. We also thank the IMU-BILTAM core facility for providing access to the Multimode Spectrophotometer and IMU Experimental Animal Facility staff for their assistance. This study is funded by grants from Scientific and Technological Research Council of Turkiye (TUBITAK) (grant no. 118S351) to B.Y., Istanbul Medeniyet University Scientific Research Projects Coordination Office (IMU-BAP 2025-GAP-Tıp −0001) to S.K.A.

## Author contributions

B.Y. designed and supervised the project, Y.Y. and S.K.A. did *in vitro* experiments and animal studies, B.Y.B. did transcriptomic analysis, C.S. did metabolomic and lipidomic experiments, E.G.D. and H.I.S. did immunohistochemistry experiments, C.S. did transcriptomic experiment, C.M.N. and A.C. did metabolomic and lipidomic bioinformatic analysis, B.Y.B. and F.Z.S. did structural docking analysis, S.C. did seahorse experiments, M.A. helped proliferation experiments, A.K. helped animal studies, B.Y., Y.Y., and S.K.A. wrote the manuscript. All authors reviewed the manuscript.

## Declaration of interests

Authors Burcu Yucel, Yahya Yozbatiran, and Saniye Koc Ada are named inventors on a pending patent application related to small-molecule modulators of TMEM97. The compounds described in the patent application were not used in the present study.

## Declaration of generative AI and AI-assisted technologies in the writing process

During the preparation of this work, the authors used ChatGPT/OpenAI and DeepSeek to revise English language and grammar, and NotebookLM to generate images for the graphical abstract. After using these tools, the authors reviewed and edited the content as needed and take full responsibility for the content of the published article.

## STAR★Methods

### Key resources table

**Table:** 

REAGENT or RESOURCE	SOURCE	IDENTIFIER
**Antibodies**		

anti-β-actin	Cell Signaling Technology	3700S; RRID:AB_2242334
anti-TMEM97	Cell Signaling Technology	62790S
anti-SQLE	Proteintech	12544-1-AP; RRID:AB_3672543
anti-rabbit IgG	Cell Signaling Technology	7074S; RRID:AB_2099233
anti-mouse IgG	Cell Signaling Technology	7076S; RRID:AB_330924
anti-LDLR	Proteintech	10785-1-AP; RRID:AB_2281164
anti-HMGCR	Abcam	ab214018; RRID:AB_2928124
anti-HMGCS1	Abcam	ab155787; RRID:AB_1951946
anti-GST	Cell Signaling Technology	2625S; RRID: AB_490796
anti-TM7SF2	Proteintech	12033-I-AP; RRID:AB_2203934
anti-DHCR24	Cell Signaling Technology	2033S; RRID:AB_2091448
anti-SREBF-1	Proteintech	14088-1-AP; RRID:AB_3670527
anti-SREBF-2	Proteintech	14508-1-AP; RRID:AB_2194235
Total OXPHOS Human WB Antibody Cocktail	Abcam	ab110411; RRID:AB_2756818

**Bacterial and virus strains**		

LentiCRISPR v2 vector	Addgene	plasmid# 52961
B834(DE3)	Sigma	69041
AcGFP1-C1 expression vector	Addgene	Plasmid #54607
PGEX-6P-3 vector	Addgene	27-4599-01

**Chemicals, peptides, and recombinant proteins**		

NBD-Cholesterol	Invitrogen	N1148
cOmplete Tablets, mini, EDTA-free, EASYpack	Fisher	BP120-500
LDL	Lee Biosolution	360-10-0.01
LPDS	Kalen Biomedical	880100–2

**Critical commercial assays**		

Dil-LDL assay	Invitrogen	L3482
FuGENE transfection reagent	Promega	E2311
DAB substrate	Thermo Scientific	34001
Amplex™ Red Cholesterol Assay Kit	Invitrogen	A12216
CellTiter-Glo® Luminescent Cell Viability Assay	Promega	G7573
One-step TUNEL *in situ* Apoptosis Kit	Elabscience	E-CK-A324
One-Glo Luciferase Reporter Assay	Promega	E−6110

**Experimental models: Cell lines**		

HEK293	ATCC	CRL-1573
HGC-27	A gift from the Department of Medical Genetics at Yeditepe University	CVCL_1279
AGS	A gift from the Department of Medical Genetics at Yeditepe University	CRL-1739
HEK293T	ATCC	CRL-3216
MKN45	A gift from the Department of Medical Genetics at Yeditepe University	CVCL_0434
HGC-27 TMEM97 KO1, HGC-27 TMEM97 KO1,	This paper	

**Experimental models: Organisms/strains**		

Mouse: Athymic Nude mouse	IMU-DEHAL	8 weeks old-BALB/c-nu (male+female)

**Oligonucleotides**		

qRT-PCR primers see Table S1	This paper	N/A
gRNAs for the TMEM97 gene see Table S2	This paper	The UCSC Genome Browser and crispr.mit.edu tools
ΔCARC TMEM97 Forward Primer 1: GCCCCCAGCCTGGTTTTTTCAGCTGCCTTTCT 2. GAAGCTGCAAGTGGATTCACACCATGACAACCT	This paper	Macrogen
ΔCARC TMEM97 Reverse Primer: 1. AGAAAGGCAGCTGAAAAAACCAGGCTGGGGGC 2. AAGGTTGTCATGGTGTGAATCCACTTGCAGCTTC	This paper	Macrogen

**Recombinant DNA**		

Acgfp c1-TMEM97	This paper	Cloned from T97cDNA

**Software and algorithms**		

MetaboAnalyst	Pang Z et al.^40^	https://www.metaboanalyst.ca/
GraphPad Prism v.10.0	GraphPad Software	https://www.graphpad.com/
ImageJ (version 1.53)	National Institutes of Health	https://imagej.net/ij/
EMBOSS Fuzzpro	Van Aalst E et al.^41^	http://imed.med.ucm.es/cgi-bin/EMBOSS/emboss.pl?_action=input&_app=fuzzpro
ColabFold	Mirdita M et al.^42^	https://colab.research.google.com/github/sokrypton/ColabFold/blob/main/AlphaFold2.ipynb
AutoDock	Eberhardt J et al.^43^	https://vina.scripps.edu/
LipidSearch v5.1	Thermo Scientific	https://www.thermofisher.com/tr/en/home/industrial/mass-spectrometry/liquid-chromatography-mass-spectrometry-lc-ms/lc-ms-software/multi-omics-data-analysis/lipid-search-software.html

**Deposited data**		

Raw and analyzed RNA-seq data	This paper	GEO: GSE312758 (NCBI)

### Experimental model and study participant details

#### Cell lines

In this study, AGS, HGC27, HEK293, and HEK293-T cell lines were cultured in high-glucose DMEM (Gibco) supplemented with 10% FBS, 1% penicillin/streptomycin, and 2 mM L-glutamine. The MKN45 cell line was cultured in RPMI 1640 (Gibco) medium containing the same supplements. All cells were incubated at 37°C in a humidified atmosphere with 5% CO_2_. AGS and HGC27 cell lines were authenticated by STR analysis. HEK293 and HEK293T cell lines were obtained from ATCC and maintained according to the recommendations. All cell lines were routinely tested for mycoplasma contamination.

#### Mouse models

The experimental design was conducted in accordance with a protocol approved by the Institutional Animal Care and Use Committee at Istanbul Medeniyet University (Approval No: 2020/2-2). A total of 24 nude mice (6-8 weeks old) were used, with 8 males in each *TMEM97* knockout group (sgTMEM97-1 and sgTMEM97-2) and 8 control mice (4 males and 4 females) in the sgCTRL group. Animals were allocated to experimental groups irrespective of sex. Mice were housed under standard specific pathogen-free conditions with *ad libitum* access to food and water in a room with controlled temperature and humidity.

### Method details

#### Immunoblotting

Western blot experiments were performed as previously described.^19^ Briefly, cells were lysed in RIPA buffer supplemented with a protease inhibitor cocktail (Roche cOmplete™ Mini, EDTA-free EASYpack). Protein concentrations were determined using the BCA assay (Thermo Scientific). A total of 16–20 μg of protein was separated by %12 SDS-PAGE, and transferred onto a PVDF membrane. Following the blocking, membranes were incubated overnight at 4°C with the following primary antibodies: TMEM97 (NOVUS, NBP1-30436), β-Actin (Cell Signaling, 8H10D10), SQLE (Proteintech, 12544-1-AP), HMGCR (Abcam, ab214018), SREBF1 (Proteintech, 14088-1-AP), SREBF2 (Proteintech, 14508-1-AP), and LDLR (Proteintech, 10785-1-AP). The next day, membranes were incubated with HRP-conjugated secondary antibodies (Cell Signaling, 7074 or 7076) for 1 h at room temperature. Blots were developed using an ECL reagent (Thermo Scientific) and visualized with an Azure C300 imaging system.

#### NBD-cholesterol uptake

Cells (2.5 × 10^4^/mL) were seeded into 96-well plates in two sets for fluorescence measurement and microscopy. They were cultured in phenol red-free DMEM/F-12 medium (Gibco) containing 10 mM glucose, 10% FBS, 1% penicillin/streptomycin, and 1% L-glutamine at 37°C with 5% CO_2_. After 24 h, the medium was replaced with either 10% FBS or 10% LPDS-containing medium for another 24 h. NBD-Cholesterol (2 mM stock) was added to a final concentration of 10 μM and incubated for 24 h. Fluorescence intensity was then measured directly using a BioTek multimode plate reader (excitation: 469 nm, emission: 537 nm). For microscopy, cells were washed with PBS, fixed with 4% paraformaldehyde for 20 min, permeabilized with 0.2% Triton X-100, stained with DAPI, and imaged using a Zeiss inverted fluorescence microscope.

#### Xenograft model

Each mouse received a subcutaneous right flank injection of 1 × 10^6^ cells mixed with Matrigel (Corning® Matrigel® Matrix, Cat. No. 354234). Following the injection, each mouse was weighed using a precision scale, and the following data were recorded; date, body weight (g), animal group (sgCTRL, sgTMEM97-1, and sgTMEM97-2). Following 1 week of matrigel degradation, animals were weighed twice weekly, and tumor size was measured using a caliper.

#### CellTiter-Glo cell viability assay

Cells were counted in serum-free DMEM and then seeded in media supplemented with 10% FBS or 10% LPDS. 200 μL suspension (25 × 10^3^ cells/mL) was added to each well of a 96-well plate with four technical replicates per condition (HGC27 sgCTRL, sgTMEM97-1, sgTMEM97-2). Plates were incubated for 4 days at 37°C with 5% CO_2_, and cell viability was measured using the CellTiter-Glo Luminescent Assay (Promega) on a BioTek multimode plate reader.

#### qRT-PCR

Total RNA was isolated using the RNeasy Plus Mini Kit (Qiagen). RNA quality and concentration were assessed using a NanoDrop spectrophotometer. cDNA was synthesized from 1 μg of RNA. For qRT-PCR, cDNA samples were diluted 1:8 in nuclease-free water, and each 20 μL reaction contained 10 μL of PowerUp SYBR Green Master Mix. The RPLP0 gene served as the housekeeping gene. qRT-PCR was performed on a Qiagen Rotor Gene Q device following the manufacturer’s protocol. Primer sequences are listed in Table 1.

#### Dil-LDL

In a 12-well plate, 150,000 cells were seeded in each well with 2 mL of DMEM supplemented with 10% FBS, with three replicates per condition, and incubated for 24 hours at 37°C in a humidified incubator containing 5% CO_2_. The cells were then washed with 500 μL of PBS and subsequently incubated for 6 hours at 37°C in 500 μL of DMEM supplemented with 10% LPDS. Following this incubation, cells were treated with 5 μg/mL Dil-LDL, and LDL uptake was visualized under a fluorescence microscope. After imaging, cells were lysed with RIPA buffer and total protein extraction was applied. Protein concentration was determined with BCA assay as described above.

#### Total cholesterol assay

Total cholesterol measurements were performed using the Amplex Red Cholesterol Assay kit (Invitrogen) according to the manufacturer’s protocol. For this experiment, TMEM97 knockout and control HGC27 cells were cultured in 12-well plates (20,000 cells/mL) in DMEM supplemented with 10% FBS, 10% LPDS, or 10% LPDS + 50 μg/mL LDL for 72 h at 37°C with 5% CO_2_. Following cell lysis and total protein isolation, the total cholesterol content was normalized by dividing it by the total protein concentration. The fluorescence intensity was measured at 560 nm (excitation) and 590 nm (emission) using a BioTek multimode plate reader.

#### Generation of HGC27 KO cell lines

To generate TMEM97 knockout (KO) HGC27 cell lines, two different guide RNAs (gRNAs) targeting the TMEM97 gene were designed using the UCSC Genome Browser and crispr.mit.edu tools (Table S2). The gRNA sequences were cloned into the LentiCRISPR v2 vector (Addgene plasmid# 52961) to obtain *TMEM97* CRISPR/Cas9 KO plasmid. Lentivirus was produced using the XtremeGene9 transfection reagent. For transduction, HGC27 cells (3×10ˆ5) were seeded in a 6-well plate and incubated overnight to reach 60-70% confluency. Virus was added to the cells at a MOI:0.5 along with 8 μg/mL polybrene. Puromycin selection (final concentration: 2 μg/mL) was applied for 48 hours post-transduction, and cells were incubated for 3 days. The efficiency of transduction was confirmed by microscopic examination. Single-cell cloning was performed to obtain monoclonal KO lines. *TMEM97* knockout was confirmed by Western blot and Sanger sequencing, identifying clones with loss of TMEM97 protein and frameshift mutations.

#### Generation of wild type and ΔCARC TMEM97 expression plasmids

*TMEM97* cDNA was obtained with PCR from MCF7 cell line total cDNA using following primer pairs forward: ATGGATCCGCCACCATGGGGGCTCCGGCAACCAGG, reverse: GCGAATTCCTTTTTTTTTTCTTTTCTCTTC. Purified PCR products cloned into PGEX-6P-3 vector, linearized with BamhI and EcoRI. ΔCARC *TMEM97* cDNA was generated with overlap PCR using following primers Forward 1: GCCCCCAGCCTGGTTTTTTCAGCTGCCTTTCT, Reverse 1: AGAAAGGCAGCTGAAAAAACCAGGCTGGGGGC, Forward 2: GAAGCTGCAAGTGGATTCACACCATGACAACCT, Reverse 2: AAGGTTGTCATGGTGTGAATCCACTTGCAGCTTC. Clonings were confirmed with diagnostic PCR and Sanger sequencing (Figure S7).

#### NBD-cholesterol binding assay

The wild type and ΔCARC *TMEM97* expression plasmids were transformed into B834(DE3) competent cells. A single colony was grown in LB medium containing kanamycin at 37°C to an OD_600_ of 0.4–0.6, induced with IPTG, and incubated overnight at 18°C. Cells were harvested by centrifugation (4000 × g, 10 min, 4°C), lysed on ice, and sonicated (50% amplitude, 3 × 10 s pulses). Lysates were purified using the Pierce™ GST Spin Purification Kit (Thermo Scientific). Purity of GST-tagged TMEM97 was verified by immunoblotting. Protein concentrations were determined with the BCA assay. For cholesterol binding, 1 μM TMEM97 was incubated with 500 nM NBD-cholesterol (Invitrogen, N1148) in PBS, and fluorescence was kinetically monitored (Ex/Em 480/540 nm) at 37°C for 1 h using a BioTek Cytation 5 reader.

#### Luciferase reporter assay

To evaluate the transcriptional activity of the *TMEM97* promoter, a dual-luciferase reporter assay was performed using the Nano-Glo® Luciferase Reporter Assay System (Promega, USA) according to the manufacturer’s instructions. Briefly, the promoter region of the *TMEM97* gene was cloned into the pGL3-promoter luciferase reporter vector (E176, Promega). HeLa cells were seeded into white, opaque 96-well plates at a density of 3×10^4^ cells per well and allowed to adhere overnight. Cells were then transiently co-transfected with the generated *TMEM97* promoter constructs (-590_+11, -340_+11, and -140_+11) or the empty vector (pGL3-promoter), alongside a pGL3-control vector (E1741, Promega) as an internal normalization control, using FuGENE HD (Promega). Following a: 24-h incubation, the culture medium was replaced with fresh medium containing either 10% Fetal Bovine Serum (FBS) or 10% Lipoprotein-Depleted Serum (LPDS) to simulate lipid-depleted conditions. After an additional 24h of treatment, luciferase activities were measured using One-Glo® Luciferase Assay (Promega) on a multi-mode microplate reader (BioTek Cytation 5 multimode plate reader, Agilent).

#### Immunoprecipitation

The *TMEM97* gene was cloned into the AcGFP1-C1 expression vector and transfected into HEK293 cells using Fugene HD (Promega). Cells were lysed in RIPA buffer supplemented with protease inhibitors and centrifuged at 12,000 rpm for 15 min at 4°C, and the resulting supernatants were collected. Co-immunoprecipitation was carried out using the GFP-Trap® system (ChromoTek), according to the manufacturer’s instructions. Lysates were incubated with equilibrated GFP-Trap beads for 1 h at 4°C, washed four times with wash buffer. Bound proteins were eluted with 200 mM glycine-HCl (pH 2.5), neutralized with 1 M Tris-HCl (pH 10.4), and analyzed by immunoblotting.

#### LC-MS analysis

Methanol/chloroform extraction was carried, polar and organic/lipid metabolites were carefully collected. Speed-vacuum drying was performed to evaporate the solvents. Polar extract was reconstituted in water: organic extract in acetonitrile/isopropanol/water (65:30:5, v/v/v). Analysis was conducted using an Exploris 240 orbitrap mass spectrometer equipped with an adjustable heated electrospray ionization (H-ESI) probe and coupled with Vanquish Flex UHPLC System (Thermo Fisher Scientific). Organic/lipid extracts were separated on an Hypersil Gold C18 2.1 mm × 100 mm × 1.9 μm particle size column. The full MS scan was acquired as such; 60,000 resolution, 65 ms maximum injection time, 200–1,450 m/z scan range. The data-dependent MS/MS scans were acquired as such; number of dependent scans 8 with 30,000 resolution, 75 ms maximum injection time, 30 V HCD collision energy. Polar extracts were separated into SeQuant ZIC-pHILIC 5 μm polymeric 150 × 2.1 mm column equipped with a 2.1 × 20 mm SeQuan ZIC-pHILIC Guard Kit. The full MS scan was acquired as such; 60,000 resolution, 100 ms maximum injection time, 80–1,200 m/z scan range. The data-dependent MS/MS scans were acquired as such; number of dependent scans 4 with 30,000 resolution, 54 ms maximum injection time, HCD collision energies of 30%, 50% and 150%.

#### Lipidomics data analysis

Lipid IDs were mapped to RefMet from the Metabolomics Workbench. Using this data, we compiled all KEGG pathways in which the mapped lipids participate. The mean fold change for these pathways was calculated by averaging the log2 fold changes of the participating lipids, and a heatmap was generated to visualize the results.

#### Seahorse assay for metabolic analysis

Cells were seeded in Seahorse XFe96 plates. After 24 hours, the medium was replenished with either DMEM or LPDS containing media. On the assay day, cells were incubated in Seahorse base medium without CO_2_ for 1 hour. The Mito Stress Test medium contained glucose, pyruvate, and glutamine, while the Glycolysis Stress Test medium contained only glutamine. OCR and ECAR were measured following sequential injections of specific modulators. All data were normalized to cell count (determined by DAPI staining) and are presented as pmol/min/10^4^ cells for OCR and mpH/min/10^4^ cells for ECAR.

#### H&E and immunohistochemistry

Sections (4 μm) from paraffine embed tissues were taken and incubated in a 60°C oven overnight. After deparaffinization with xylene, the sections were rehydrated and subjected to hematoxylin & eosin (H&E) staining or immunohistochemistry (IHC). Slides were imaged on Olympus BX61 microscope equipped with a DP72 digital camera, and images were analyzed with QuPath.^44^ IHC staining was performed by using SensiTek HRP Anti-Polyvalent Lab Pack (ScyTek Laboratories, SHP125). Briefly, sections were blocked in 3% hydrogen peroxide and subjected to antigen retrieval by soaking slides in Tris-EDTA buffer and heating to 95°C for 20 min. After blocking, the sections were incubated with Ki67 (orb389335), SQLE (1:200), SREBF1 (1:200), SREBF2 (1:200), LDLR (1:200), and HMGCR (1:200) antibodies at 4°C, overnight. Biotinylated secondary antibody and streptavidin/HRP were applied at room temperature for 20 min. Ki67 evaluation was done by counting Ki67 positive cells in five randomly selected areas and dividing it by the total cell number.^45^ Other IHC stainings were evaluated by using H-score method in the mantle zone of the tumors. The necrotic area was determined using H&E-stained sections and expressed as a ratio of the necrotic area to the total tumor area.

#### TUNEL assay

Apoptotic cells were detected using the One-step TUNEL *In Situ* Apoptosis Kit (Elabscience, E-CK-A320) following the manufacturer’s instructions. Briefly, the dewaxed sections were washed with PBS and incubated with Proteinase K at 37°C for 20 min. Labeling was achieved by FITC labelling solution at 37°C for 60 min. After DAPI counterstaining, slides were mounted with coverslips and imaged under the Olympus BX61 fluorescence microscope. The apoptotic index was calculated by dividing the number of apoptotic cells in five randomly selected areas by the number of total cells.^46^

#### Preparation and sequencing of RNA-Seq libraries

100 ng of total RNA input was subjected to library construction with the Illumina Stranded Total RNA Prep, Ligation with Ribo-Zero Plus kit (Illumina). The adapter-ligated cDNA fragments were purified using AMPure beads (Beckman-Coulter) and subjected to PCR amplification to add unique dual Illumina DNA/RNA UD Indexes (Illumina) and primer sequences for cluster generation and sequencing. The obtained dual-indexed libraries were quantified using Qubit dsDNA BR Assay kit (Invitrogen). Pooled libraries were sequenced with S1 Reagent Kit v1.5 (300 cycles) at 1 nM starting concentration using an Illumina NovaSeq6000 platform with NovaSeq 6000 in 2 × 151 bp pair-end sequencing mode, achieving an average depth of 32 million reads per sample.

#### RNA seq data processing

The *TMEM97* gene was knocked out using two independent sgRNAs (SG1 and SGctrl) under FBS and LPDS conditions, with corresponding controls (SGCTRL-FBS and SGCTRL-LPDS). Each condition was analyzed in three biological replicates. All samples were processed and sequenced within the same flow cell to minimize batch effects. Raw BCL files were converted to FASTQ format using bcl2fastq. Quality control and trimming were performed with Sickle, retaining reads with a minimum Phred quality score of 28 and a minimum length of 100 bp to ensure selection of high-quality mRNA reads. Reads were aligned to the human reference genome (hg38) using STAR aligner with the corresponding GTF annotation file. Gene-level read counts were generated using HTSeq-count without prior normalization. Differential expression analysis was performed in DESeq2.^47^ Given that all samples were run on the same flow cell, batch correction was not applied. Normalization for library size differences was performed using median of ratios method implemented in DESeq2. Functional enrichment was done with g:Profiler (KEGG, Reactome, WikiPathways),^48^ and cholesterol metabolism–related genes from KEGG were compared against DESeq2 results. A heatmap was generated to visualize expression patterns of these cholesterol-related genes.

#### Motif analysis

To investigate potential cholesterol-binding regions in the human TMEM97 protein, sequence-based motif scanning was performed using EMBOSS Fuzzpro.^41^ The human TMEM97 protein sequence was retrieved from UniProt, and the search targeted canonical cholesterol-recognition motifs: CARC and CRAC. The analysis identified four CARC and three CRAC motifs within the sequence, of which two CARC motifs were located within predicted transmembrane (TM) domains (Table S3). TM domain prediction was used to contextualize motif positions with respect to membrane topology.

#### Protein structure modeling

As no experimental structure of human TMEM97 is available in the PDB, the full-length sequence was modeled *de novo* using ColabFold (AlphaFold2 implementation).^42^ The modeling parameters were set as follows: num_relax=5, template_mode=none, num_recycles=48, relax_max_iterations=200, and pairing_strategy=greedy. No structural templates were used. The modeled human TMEM97 structure showed approximately 78% sequence identity and high structural similarity to the bovine homolog.

#### Molecular docking

Cholesterol binding to the modeled human TMEM97 structure was evaluated using AutoDock.^43^ Docking simulations were conducted using a blind docking approach across the entire protein surface, with particular emphasis on the four CARC and three CRAC motifs, as well as a predicted binding pocket region. A total of 100 docking runs were performed (5 replicates × 20 runs each) to ensure sampling consistency. Of the seven motifs, amino acids between 97–105 were located within predicted transmembrane helices and showed strong cholesterol binding signals.

#### Functional validation via deletion mutagenesis

To assess the functional relevance of the 97–105 region, this region was deleted and the truncated TMEM97 structure was remodeled in ColabFold using the same parameters as above. The new structure was subjected to cholesterol docking using the same blind protocol. Subsequent docking showed that cholesterol no longer bound the original site but instead occupied a new cavity formed by the deletion, indicating disruption of the binding interface.

#### Conservation analysis

To evaluate evolutionary conservation of the 97–105 region, BLASTP analysis was performed against a range of vertebrate species. The results indicated a high degree of sequence conservation in this region, supporting its potential functional relevance in cholesterol binding.

### Quantification and statistical analysis

Metabolite identification and relative quantification were performed using Compound Discoverer v3.3, and lipid profiling with LipidSearch v5.1 (Thermo Scientific). Pathway enrichment analysis and direct pathway mapping were performed using the MetaboliticsDB tool.^40^ For metabolite set enrichment analysis, we used MetaboAnalyst.^40^ To generate volcano plots, the Metabolomics Workbench was used.^49^ All *in vitro* experiments were performed in three independent experiments with at least in triplicates except Seahorse analysis (four technical replicates in two independent experiments). RNAseq and metabolomic analysis data obtained from single run with three independent biological samples in each group. Statistical details of all experiments, including exact *n* values and the tests applied, can be found in the figure legends and Results section. Statistical analyses were performed using GraphPad Prism (v10.0, GraphPad Software) and ImageJ (v1.53). Statistical analyses were performed using Welch’s t-test. Statistical significance was defined as *p* < 0.05 and is indicated as follows: ∗*p* < 0.05; ∗∗*p* < 0.01; ∗∗∗*p* < 0.001; ∗∗∗∗*p* < 0.0001. Unless otherwise stated, data are presented as mean ± SD.

## Supplemental information

Document S1. Figures S1–S7, Tables S1–S3, and Data S1

Data S2. H-score quantification of xenograft tumor sections by immunohistochemical analysis
