# Supplementary material for: TMEM97 regulates cholesterol biosynthesis and mitochondrial metabolism in gastric carcinoma
Source: iScience. 2026 Jul 14;29(8):116773. doi: 10.1016/j.isci.2026.116773 (PMC13382799; doi:10.1016/j.isci.2026.116773)
Supplement: Document S1. Figures S1–S7, Tables S1–S3, and Data S1 [file mmc1.pdf]

iScience, Volume *29*

Supplemental information

TMEM97 regulates cholesterol biosynthesis

and mitochondrial metabolism in gastric carcinoma

Saniye Koç Adą, Yahya Yozbatıran, Bengü Yaren Beyaz, Ceren Sarı, Elif Gelenli Dolanbay, Halil İbrahim Saygı, Ceren Sümer, Cyrille Mesue Njume, Ali Çakmak, Fatma Zehra Sarı, Şeyma Çimen, Mertkaya Aras, Andaç Kılıçkap, and Burcu Yücel

Supplementary Figures

Figure S1

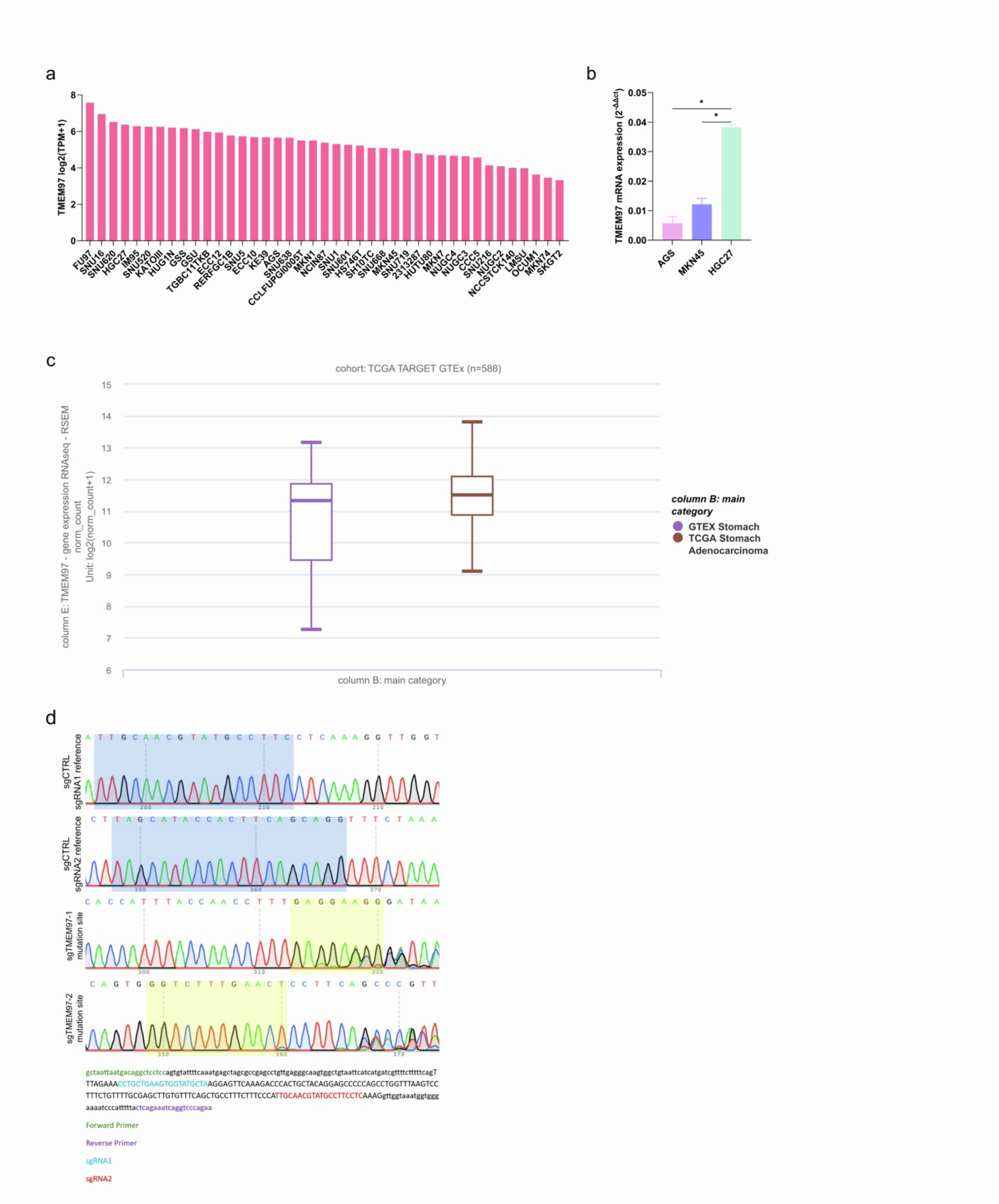

Figure S1. TMEM97 expression profiling across gastric cancer models and impact on necrosis.

a TMEM97 mRNA expression levels across 37 human gastric cancer cell lines, based on RNA sequencing data from the DepMap Expression Public 24Q4 dataset. Expression values are shown as $\log_2$(TPM + 1), indicating that TMEM97 is highly expressed in a subset of gastric cancer cell lines.

b TMEM97 mRNA expression levels in AGS, MKN45 and HGC27 cell lines**.** Statistical significance was determined using Welch's t-test and is indicated as follows: *$P < 0.05$; **$P < 0.01$. Data are presented as mean ± SD.

c Boxplot comparison of TMEM97 expression between normal stomach tissues (GTEx) and stomach adenocarcinoma samples (TCGA), obtained via UCSC Xena Browser. Expression values are RSEM-normalized and presented as $\log_2$(norm_count + 1).

d Validation of TMEM97 knockout in HGC27 cell lines by Sanger sequencing. Comparison of Sanger sequencing chromatograms from TMEM97-KO cell lines generated using a control group and two different sgRNAs (sgTMEM97-1 and sgTMEM97-2).

Figure S2

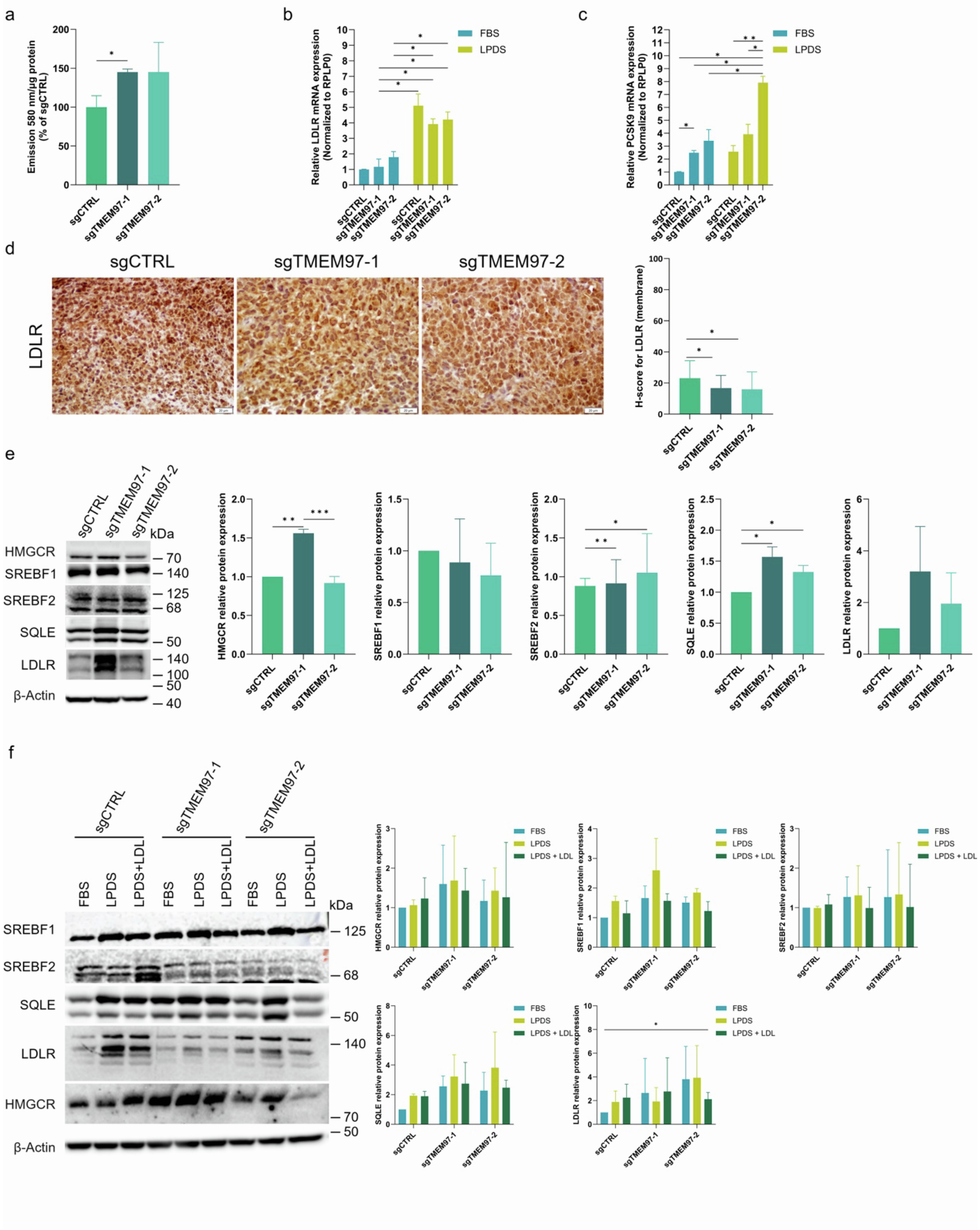

a
Emission 580 nm/µg protein (% of sgCTRL)
sgCTRL
sgTMEM97-1
sgTMEM97-2
b
Relative LDLR mRNA expression (Normalized to RPLP0)
FBS
LPDS
c
Relative PCSK9 mRNA expression (Normalized to RPLP0)
d
sgCTRL
sgTMEM97-1
sgTMEM97-2
LDLR
H-score for LDLR (membrane)
e
HMGCR
SREBF1
SREBF2
SQLE
LDLR
β-Actin
kDa
HMGCR relative protein expression
SREBF1 relative protein expression
SREBF2 relative protein expression
SQLE relative protein expression
LDLR relative protein expression
f
FBS
LPDS
LPDS+LDL
LPDS + LDL

Figure S2. TMEM97 knockout induces SREBP-mediated compensatory pathways in gastric carcinoma cells under lipoprotein-depleted conditions.

a Dil-LDL uptake is elevated in TMEM97 knockout HGC27 cell lines compared to sgCTRL, indicating enhanced LDLR-mediated endocytosis following TMEM97 loss.

b-c RT-qPCR analysis of LDLR **(b)** and PCSK9 **(c)** expressions between TMEM97 knockout clones under both FBS and LPDS conditions.

d Immunohistochemical staining of xenograft tumor sections showed increased cytoplasmic LDLR in TMEM97 knockout cells, indicating enhanced LDLR endocytosis. Scale bar, 20 μm.

e Western blot analysis of HMGCR, SREBF-1, SREBF-2, SQLE, LDLR, and HMGCS1 protein expression levels in TMEM97 KO cells cultured in normal medium, with β-Actin as the loading control. The uncropped versions of the immunoblots are provided in the Supplemental materials. Densitometric analysis of HMGCR, SREBF-1, SREBF-2, SQLE, LDLR, and HMGCS1 protein expression levels in sgCTRL and TMEM97-knockout HGC27 cells, normalized to β-actin. The corresponding quantitative data are presented alongside the representative Western blot bands**.**

f Western blot of SREBF-1/2, HMGCR, SQLE, and LDLR (β-Actin as loading control) in sgCTRL versus TMEM97 knockout monoclonal HGC27 cells cultured in 10% FBS, LPDS, or LPDS + LDL (50 μg/mL). The uncropped versions of the immunoblots are provided in the Supplemental materials The corresponding quantitative data are presented alongside the representative Western blot bands**.** Each band was normalized against its own beta-actin value. Statistical significance was determined by using Welch's t-test and is indicated as follows: *$P < 0.05$; **$P < 0.01$; ***$P < 0.001$. Data are presented as mean ± SD.

Figure S3

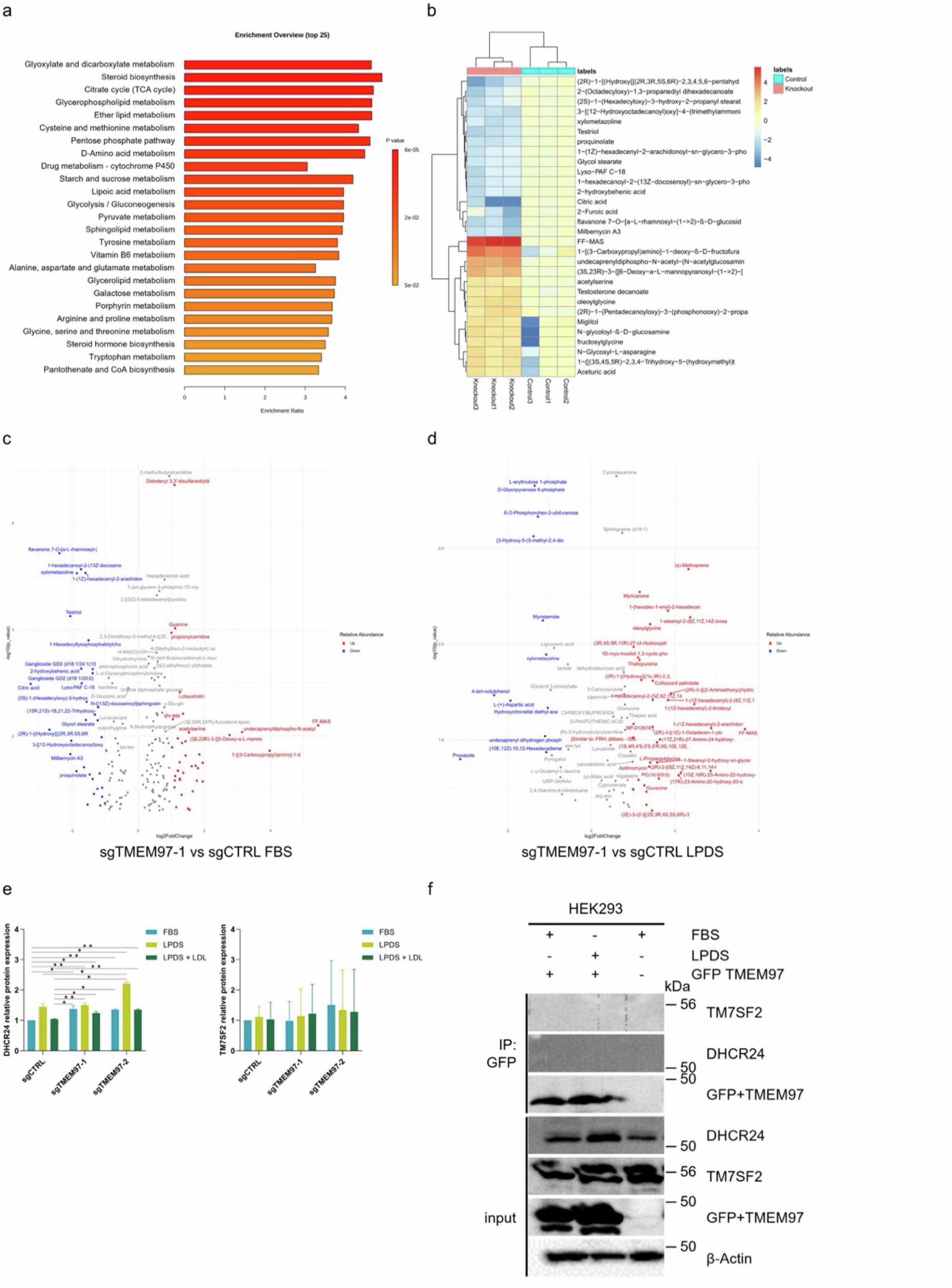
a
Enrichment Overview (top 25)
Glyoxylate and dicarboxylate metabolism
Steroid biosynthesis
Citrate cycle (TCA cycle)
Glycerophospholipid metabolism
Ether lipid metabolism
Cysteine and methionine metabolism
Pentose phosphate pathway
D-Amino acid metabolism
Drug metabolism - cytochrome P450
Starch and sucrose metabolism
Lipoic acid metabolism
Glycolysis / Gluconeogenesis
Pyruvate metabolism
Sphingolipid metabolism
Tyrosine metabolism
Vitamin B6 metabolism
Alanine, aspartate and glutamate metabolism
Glycerolipid metabolism
Galactose metabolism
Porphyrin metabolism
Arginine and proline metabolism
Glycine, serine and threonine metabolism
Steroid hormone biosynthesis
Tryptophan metabolism
Pantothenate and CoA biosynthesis
P value
6e-05
2e-02
5e-02
Enrichment Ratio
b
labels
Control
Knockout
Testriol
proquinolate
Glycol stearate
Lyso-PAF C-18
2-hydroxybehenic acid
Citric acid
2-Furoic acid
Milbemycin A3
FF-MAS
acetylserine
Testosterone decanoate
oleoylglycine
Miglitol
N-glycoloyl-ß-D-glucosamine
fructosylglycine
N-Glycosyl-L-asparagine
Aceturic acid
Knockout3
Knockout1
Knockout2
Control3
Control1
Control2
c
log2FoldChange
-log10(p_value)
Relative Abundance
Up
Down
sgTMEM97-1 vs sgCTRL FBS
d
log2FoldChange
-log10(p_value)
Relative Abundance
Up
Down
sgTMEM97-1 vs sgCTRL LPDS
e
DHCR24 relative protein expression
FBS
LPDS
LPDS + LDL
sgCTRL
sgTMEM97-1
sgTMEM97-2
TM7SF2 relative protein expression
FBS
LPDS
LPDS + LDL
sgCTRL
sgTMEM97-1
sgTMEM97-2
f
HEK293
FBS
LPDS
GFP TMEM97
kDa
56
TM7SF2
IP:
GFP
50
DHCR24
50
GFP+TMEM97
DHCR24
50
56
TM7SF2
input
50
GFP+TMEM97
50
β-Actin

Figure S3. Metabolomic profiling of TMEM97 knockout cells.

a KEGG pathway enrichment analysis of the 25 most abundant metabolites in sgTMEM97-1 versus sgCTRL cells cultured in FBS, using MetaboAnalyst.

b In cells cultured in FBS, FF-MAS was markedly elevated in sgTMEM97-1 versus sgCTRL among the top 30 differentially abundant metabolites.

c-d Volcano plots of metabolites significantly altered in sgTMEM97-1 versus sgCTRL cells under (c) FBS and (d) LPDS culture conditions. In each plot, red and blue points represent up- and down-regulated metabolites ($p < 0.05$, $|\log_2 FC| > 1$), respectively, based on relative abundance; all plots were generated via the Metabolomics Workbench.

e Densitometric analysis of the protein expression of TM7SF2 and DHCR24 in TMEM97 knockout cells under 10% FBS, LPDS, or LPDS + LDL (50 µg/mL) culture conditions. Each band was normalised against its own beta-actin value.

f GFP co-immunoprecipitation (co-IP) assay was performed in HEK293 cells overexpressing GFP-TMEM97 cultured in both FBS and LPDS medium to evaluate interaction with TM7SF2 and DHCR24. Statistical significance was determined using Welch's t-test and is indicated as follows: *P < 0.05; **P < 0.01; ***P < 0.001; ****P < 0.0001. Data are presented as mean ± SD.

Figure S4

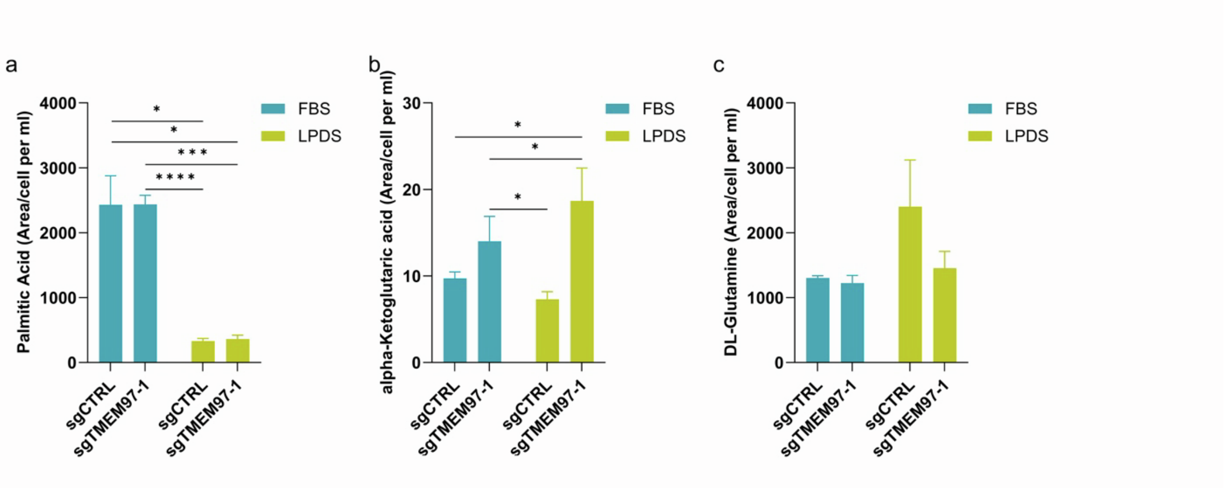

Figure S4. TMEM97 deficiency alters mitochondrial metabolites and redox-related cofactor levels.

a-c Relative abundance of mitochondrial metabolites palmitic acid **(a)**, glutamine **(b)**, and α-ketoglutaric acid **(c)** in sgCTRL and TMEM97 knockout HGC27 cells cultured in FBS or LPDS. Statistical significance was determined using Welch's t-test and is indicated as follows: $^{*}P < 0.05$; $^{**}P < 0.01$; $^{***}P < 0.001$. Data are presented as mean ± SD.

Figure S5

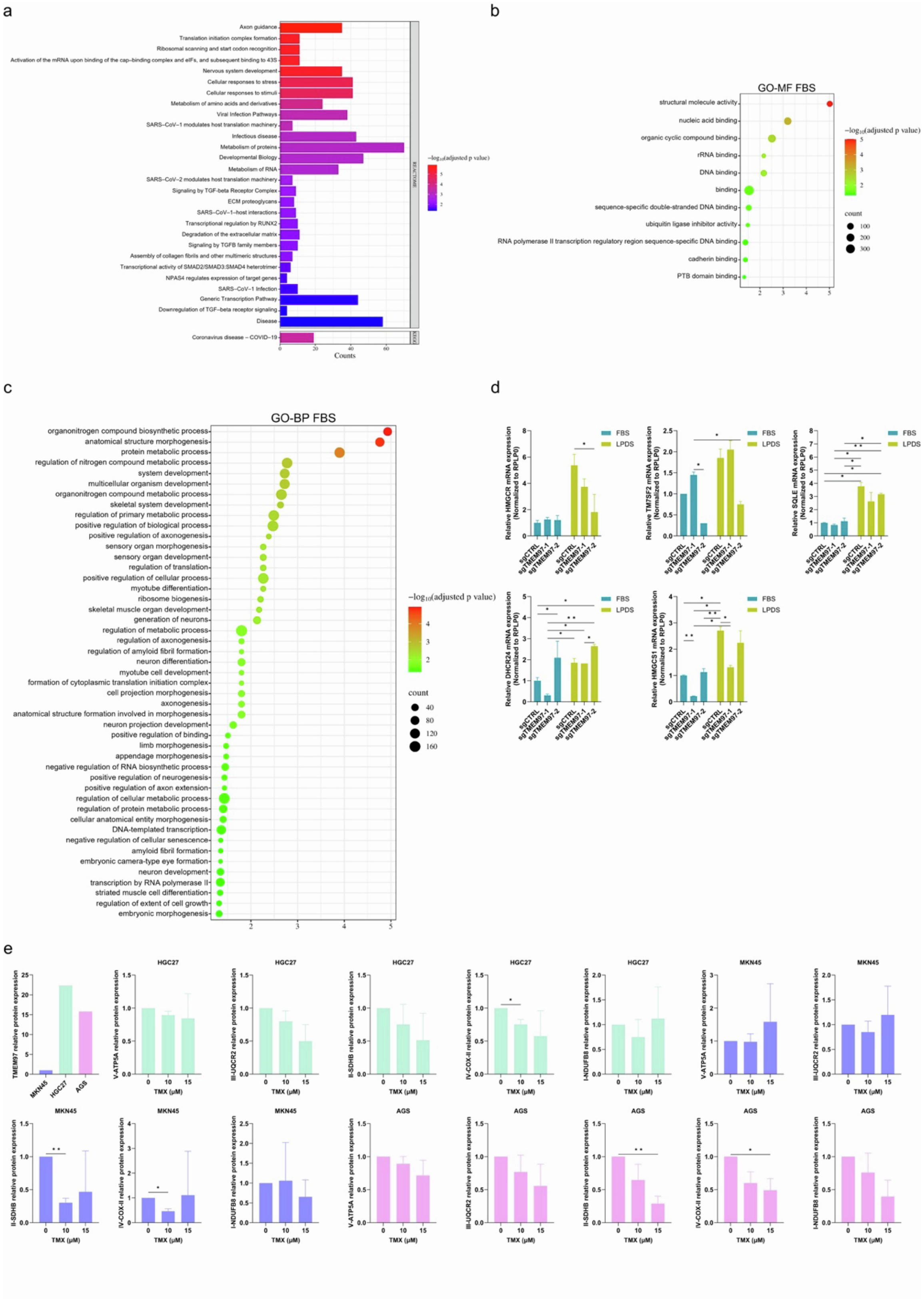

Figure S5. Transcriptomic analysis reveals environment-specific gene expression changes and preserved cholesterol biosynthesis under lipid-deprived conditions.

a Reactome-based pathway enrichment analysis in sgTMEM97-1 cells under FBS conditions.

b Gene Ontology (GO) analysis of molecular functions (GO-MF) in sgTMEM97-1 cells under FBS conditions.

c Gene ontology (GO) biological process (BP) analysis of DEGs shows transcriptional change in the main metabolic regulatory systems.

d RT-qPCR analysis of HMGCR, TM7SF2, SQLE, DHCR24 and HMGCS1 mRNA expressions under both FBS and LPDS conditions.

e-f Densitometric analysis of the Western blot of TMEM97 (Figure 5e), V-ATP5A, III-UQCR2, II-SDHB, IV-COX-II and I-NDUFB8 protein expression (OXPHOS proteins) (Figure 5f) by normalized with β-Actin in MKN45, AGS and HGC27 cells (n=3). Statistical significance was determined by unpaired Student's t-test and is indicated as follows: $*P < 0.05$; $**P < 0.01$. Data are presented as mean ± SD.

Figure S6

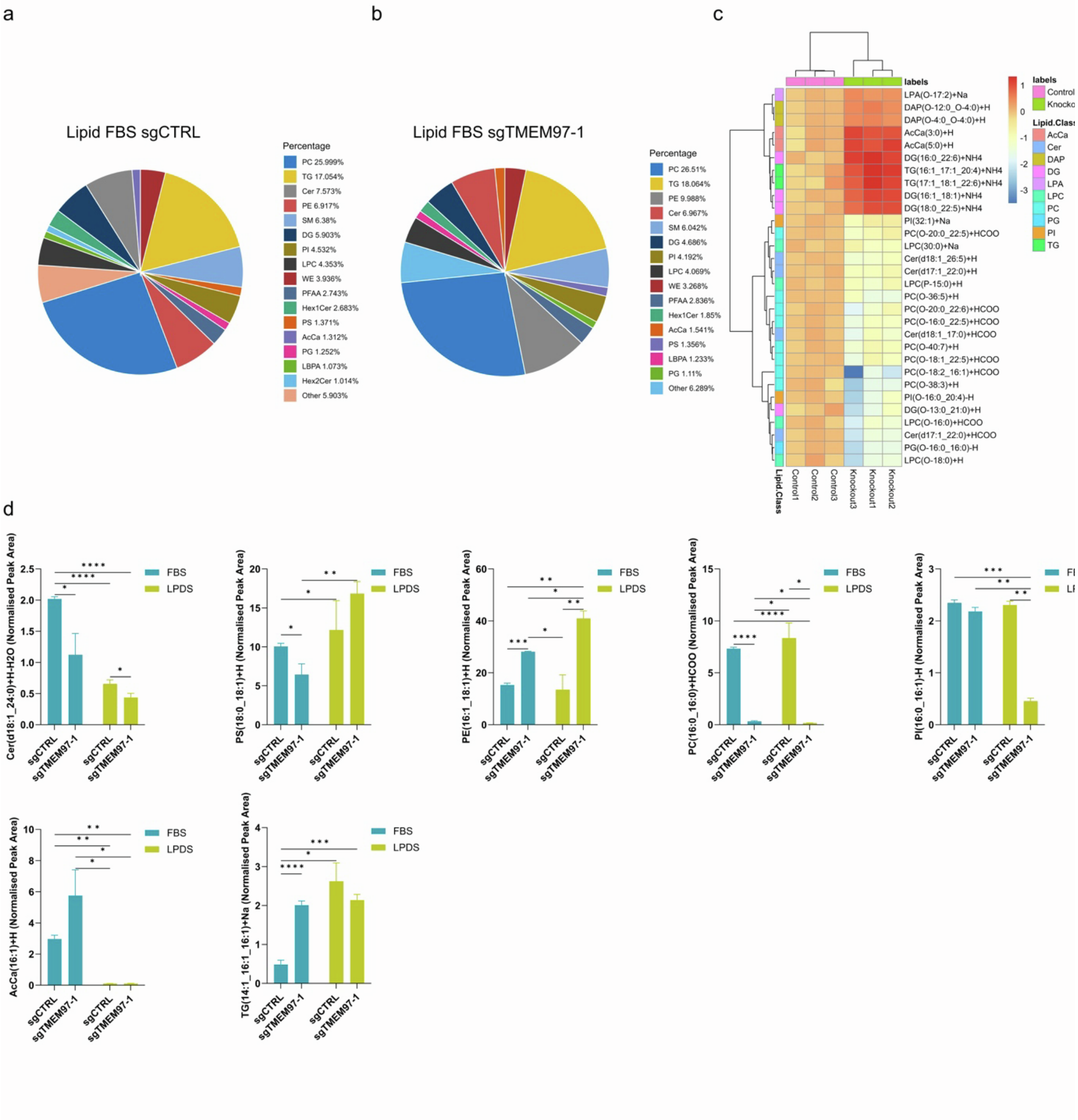

Figure S6. TMEM97 deficiency alters lipid profiling and modulates lipogenic gene expression in both normal and lipid depleted media.

a-b Pie charts displaying the distribution of lipid classes in sgCTRL (a) and sgTMEM97-1 (b) HGC27 cells cultured under FBS conditions.

c Heatmap showing the top 30 most diffirentially expressed lipid species under FBS conditions in sgCTRL and sgTMEM97-1 cells.

d Targeted quantification of selected lipid species, including ceramide (Cer), phosphatidylserine (PS), phosphatidylethanolamine (PE), phosphatidylcholine (PC), phosphatidylinositol (PI), acylcarnitine 16:1 (AcCa), and triglyceride TG(14:1_16:1_16:1) in sgCTRL and sgTMEM97-1 cells cultured either in FBS or LPDS. Statistical significance was determined using Welch's t-test and is indicated as follows: *P < 0.05; **P < 0.01; ***P < 0.001; ****P < 0.0001. Data are presented as mean ± SD.

Figure S7

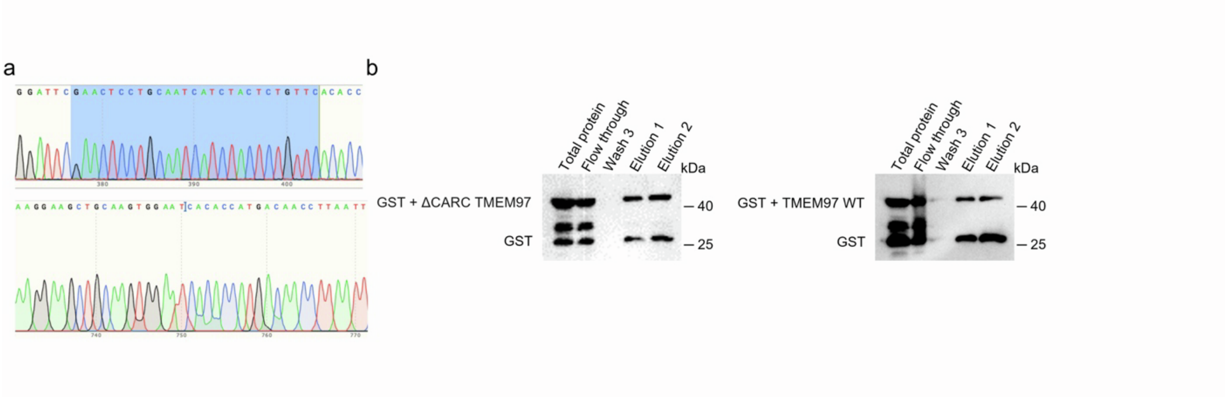

Figure S7. Validation of ΔCARC TMEM97.

a Sanger sequencing chromatogram of wild-type (top) and ΔCARC (bottom) TMEM97 constructs. The targeted deletion is confirmed by the absence of the nucleotide sequence encoding residues 97–105, highlighted in blue.

b Western blot analysis of GST-tagged wild-type and ΔCARC TMEM97 proteins after purification using GST spin columns. Total lysate, flow-through, and elution fractions (Elution 1 and Elution 2) are shown. The uncropped versions of the immunoblots are provided in the Supplemental materials.

Supplementary Tables

Table S1. qRT-PCR primers

TMEM97-F	TGCTGAAGTGGTATGCTAAGG
TMEM97-R	TGGAGAGTATCGGAATTAAGGTTG
SQLE-F	GATGCAGCTATTTTCGAGGC
SQLE-R	AGGGTTAGGAGACAATACAGAAAG
HMGCR-F	GCCATTTTGCCCGAGTTTTAG
HMGCR-R	TGCCAGAGGGAAACACTTG
HMGCS1-F	TCTCCATACAGTGCTACCTCAG
HMGCS1-R	AGTCATTCAGCAACATCCGAG
LDLR-F	ACATCTACTGGACCGACTCTG
LDLR-R	TGTTTTCAGTCACCAGCGAG
PCSK9 -F	CACAGAGTGGGACATCACAG
PCSK9-R	TTTGGCAGAGAAGTGGATCAG
Tm7sf2-F	GCTATCCTATTAACGGCTTCCA
Tm7sf2-R	GTAGAGAAAGAGGCTGAAGATGAA
DHCR24-F	GTGAAACACTTTGAAGCCAGG
DHCR24-R	CAGATCTTGTCGTACACCTCG
RPLPO-F	AGCATCTACAACCCTGAAGTG
RPLPO-R	AGCAAGTGGGAAGGTGTAATC
SCD1-F	CCCTACGGCTCTTTCTGATC
SCD1-R	GTACTCCCCTTCTCTTTGACAG
FASN-F	CTCTGGTTCATCTGCTCTGG
FASN-R	ATCAAAGGTGCTCTCGTCTG
SREBP1-F	GTCTACCATAAGCTGCACCAG
SREBP1-R	ACTGGTCTTCACTCTCAATGC
SREBP2-F	AGCCTCAACCTCAAACTCAG
SREBP2-R	AGGATGTCACCAGGCTTTG

Table S2. sgRNAs for TMEM97 gene

TMEM97 sgRNA_1F	caccGAGGAAGGCATACGTTGCAA
TMEM97 sgRNA_1R	aaacTTGCAACGTATGCCTTCCTC
TMEM97 sgRNA_2F	caccGCGCGAGCTCTACCCAGTCG
TMEM97 sgRNA_2R	aaacCGACTGGGTAGAGCTCGCGC
TMEM97 sgRNA_3F	caccGGGCGCCCCTCACCTCGACT
TMEM97 sgRNA_3R	aaacAGTCGAGGTGAGGGGCGCCC
TMEM97 sgRNA_4F	caccgCCTGCTGAAGTGGTATGCTA
TMEM97 sgRNA_4R	aaacTAGCATACCACTTCAGCAGGc
TMEM97 sgRNA_5F	caccgCATTTACCAACCTTTGAGGA
TMEM97 sgRNA_5R	aaacTCCTCAAAGGTTGGTAAATGc
control sgRNA_1F	caccGGCATCGCCCTCCTTTGTCA
control sgRNA_1R	aaacTGACAAAGGAGGGCGATGCC
control sgRNA_2F	caccgATTATACCTCTTCACAGCTG
control sgRNA_2R	aaacCAGCTGTGAAGAGGTATAATc

Table S3. CARC and CRAC sequences detected by in silico analysis

Sequence:<br>HitCount:	NP_055388.2<br>8		from:	1	to:	176
Pattern_name		Mismatch	Pattern			
pattern		0	**[RK]-X(1,5)-[YF]-X(1,5)-[LV]**			
Start	End	Pattern	Mismatch	Sequence		
97	105	pattern:[RK]-X(1,5)-[YF]-X(1,5)-[LV]	.	RTPAIIYSV		
67	74	pattern:[RK]-X(1,5)-[YF]-X(1,5)-[LV]	.	KSFLFCEL		
67	75	pattern:[RK]-X(1,5)-[YF]-X(1,5)-[LV]	.	KSFLFCELV		
52	58	pattern:[RK]-X(1,5)-[YF]-X(1,5)-[LV]	.	KEFKDPL		
52	59	pattern:[RK]-X(1,5)-[YF]-X(1,5)-[LV]	.	KEFKDPLL		

48	58	pattern:[RK]-X(1,5)-[YF]-X(1,5)-[LV]	.	KWYAKEFKDPL			
48	59	pattern:[RK]-X(1,5)-[YF]-X(1,5)-[LV]	.	KWYAKEFKDPLL			
36	41	pattern:[RK]-X(1,5)-[YF]-X(1,5)-[LV]	.	RELYPV			
Sequence:<br>HitCount:	NP_055388.2<br>15		from:	1	to:	176	
Pattern_name		Mismatch	Pattern				
pattern		0	**[LV]-X(1,5)-[YF]-X(1,5)-[RK]**				
Start	End	Pattern	Mismatch	Sequence			
162	168	pattern:[LV]-X(1,5)-[YF]-X(1,5)-[RK]	.	LRSPYYK			
162	172	pattern:[LV]-X(1,5)-[YF]-X(1,5)-[RK]	.	LRSPYYKYEEK			
162	173	pattern:[LV]-X(1,5)-[YF]-X(1,5)-[RK]	.	LRSPYYKYEEKR			
158	163	pattern:[LV]-X(1,5)-[YF]-X(1,5)-[RK]	.	LIFMLR			
157	163	pattern:[LV]-X(1,5)-[YF]-X(1,5)-[RK]	.	LLIFMLR			
119	125	pattern:[LV]-X(1,5)-[YF]-X(1,5)-[RK]	.	LFEDFSK			
115	125	pattern:[LV]-X(1,5)-[YF]-X(1,5)-[RK]	.	LSTFLFEDFSK			
47	52	pattern:[LV]-X(1,5)-[YF]-X(1,5)-[RK]	.	LKWYAK			
47	55	pattern:[LV]-X(1,5)-[YF]-X(1,5)-[RK]	.	LKWYAKEFK			
46	52	pattern:[LV]-X(1,5)-[YF]-X(1,5)-[RK]	.	LLKWYAK			
46	55	pattern:[LV]-X(1,5)-[YF]-X(1,5)-[RK]	.	LLKWYAKEFK			
41	48	pattern:[LV]-X(1,5)-[YF]-X(1,5)-[RK]	.	VEFRNLLK			
38	48	pattern:[LV]-X(1,5)-[YF]-X(1,5)-[RK]	.	LYPVEFRNLLK			
34	44	pattern:[LV]-X(1,5)-[YF]-X(1,5)-[RK]	.	LPRELYPVEFR			
33	44	pattern:[LV]-X(1,5)-[YF]-X(1,5)-[RK]	.	VLPRELYPVEFR			

Data S1.

Original uncropped western blots related to Figure 1, Figure 2, Figure 3 and Figure 5.

Figure 1a

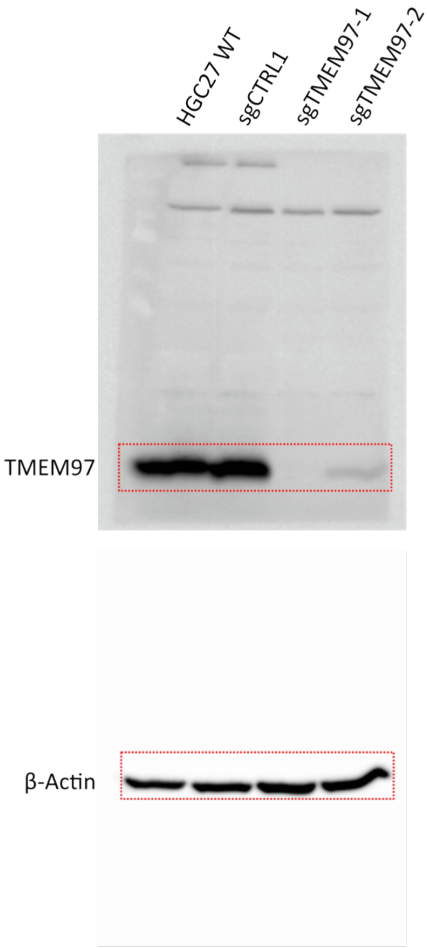

Figure 2b

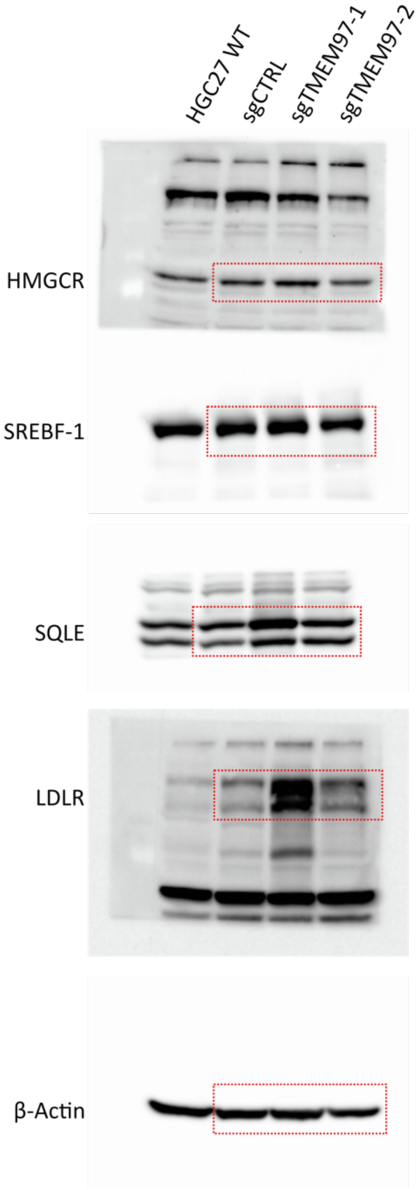
HGC27 WT
sgCTRL
sgTMEM97-1
sgTMEM97-2
HMGCR
SREBF-1
SQLE
LDLR
β-Actin

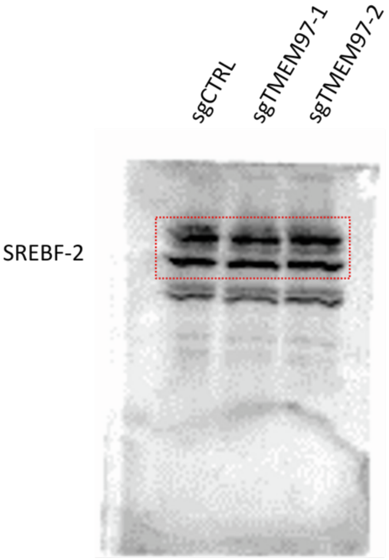
sgCTRL
sgTMEM97-1
sgTMEM97-2
SREBF-2

Figure 2d

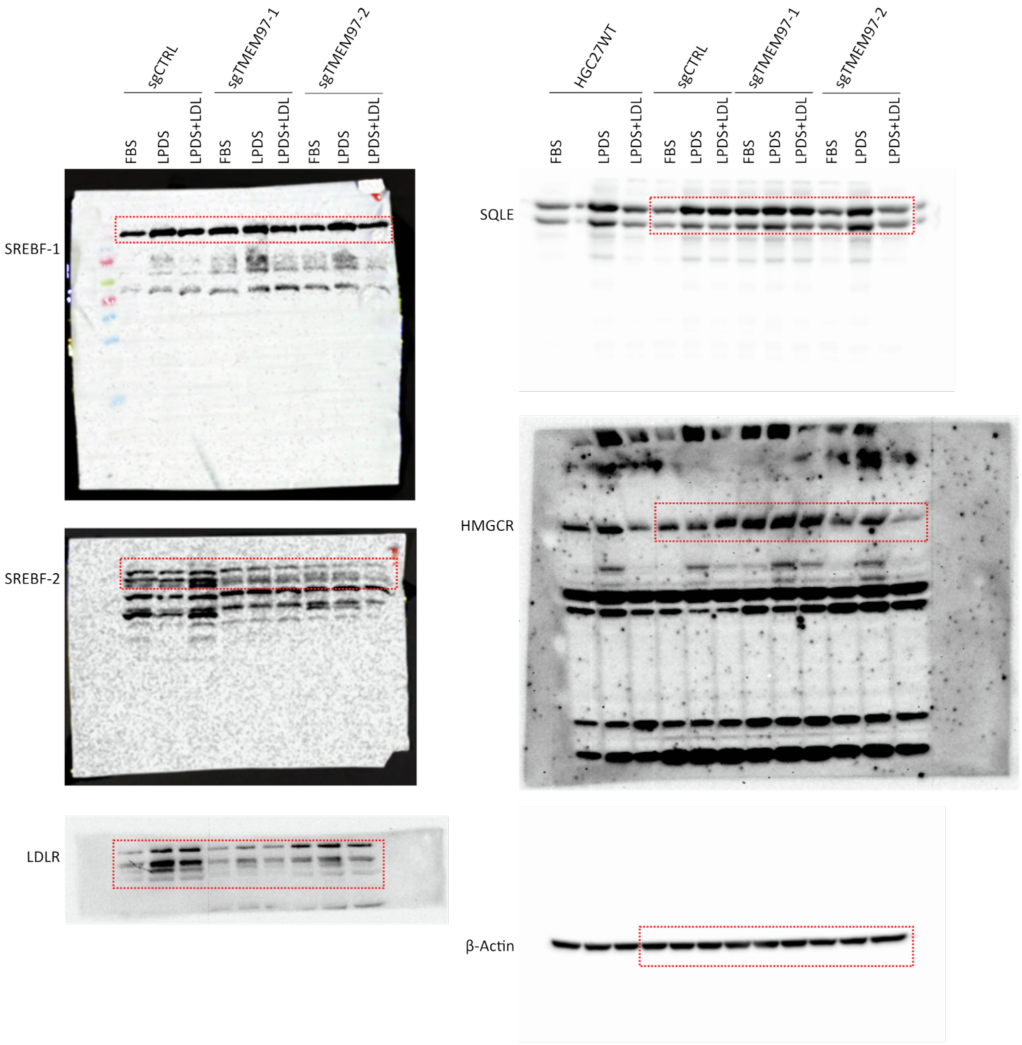
sgCTRL
sgTMEM97-1
sgTMEM97-2
FBS
LPDS
LPDS+LDL
HGC27WT
SREBF-1
SREBF-2
LDLR
SQLE
HMGCR
β-Actin

Figure 3e

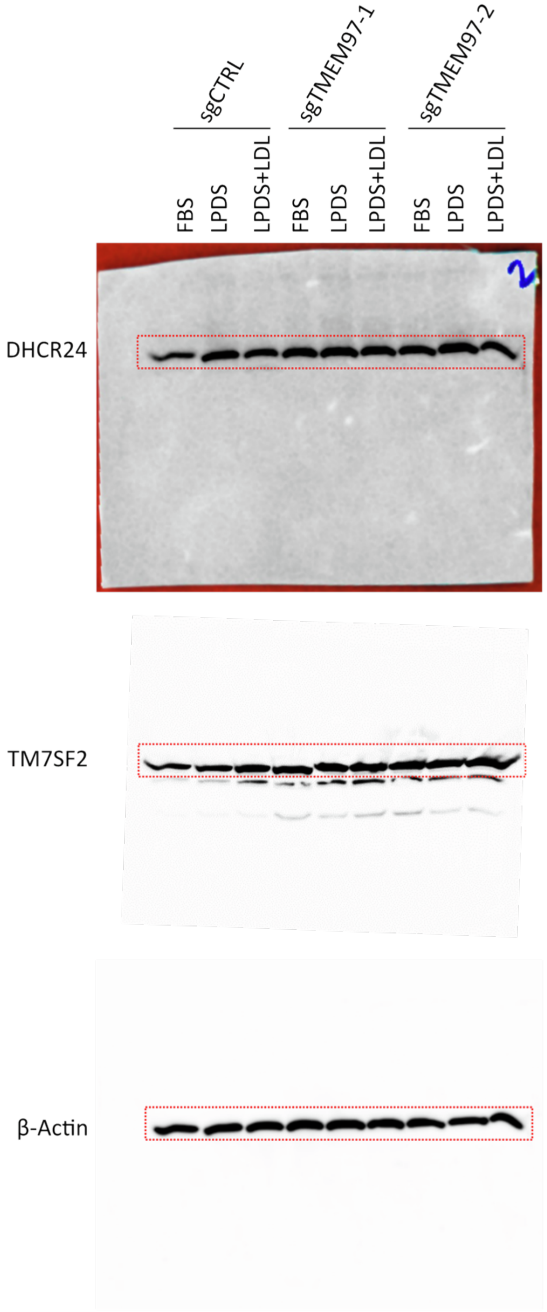

sgCTRL
sgTMEM97-1
sgTMEM97-2
FBS
LPDS
LPDS+LDL
FBS
LPDS
LPDS+LDL
FBS
LPDS
LPDS+LDL
DHCR24
TM7SF2
β-Actin

Figure 3f

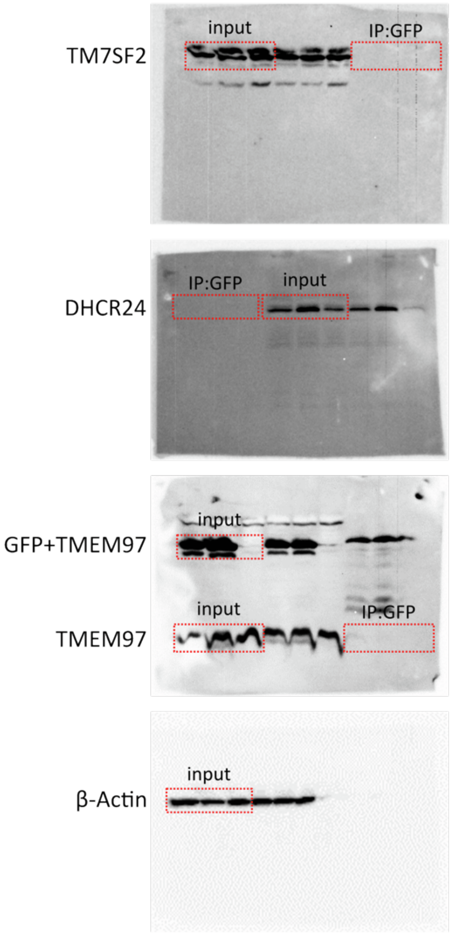
TM7SF2
input
IP:GFP
DHCR24
IP:GFP
input
GFP+TMEM97
input
TMEM97
input
IP:GFP
β-Actin
input

Figure 5e

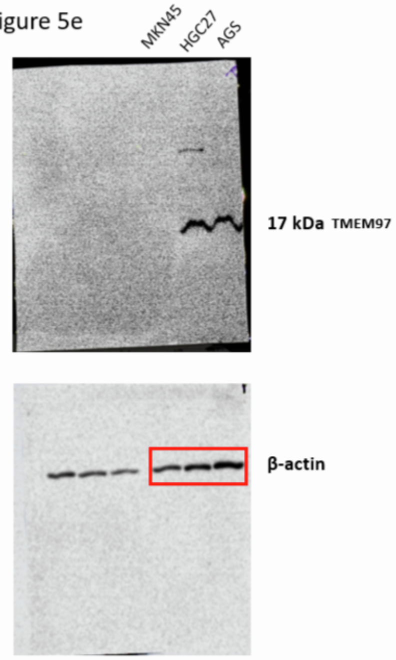

Figure 5f

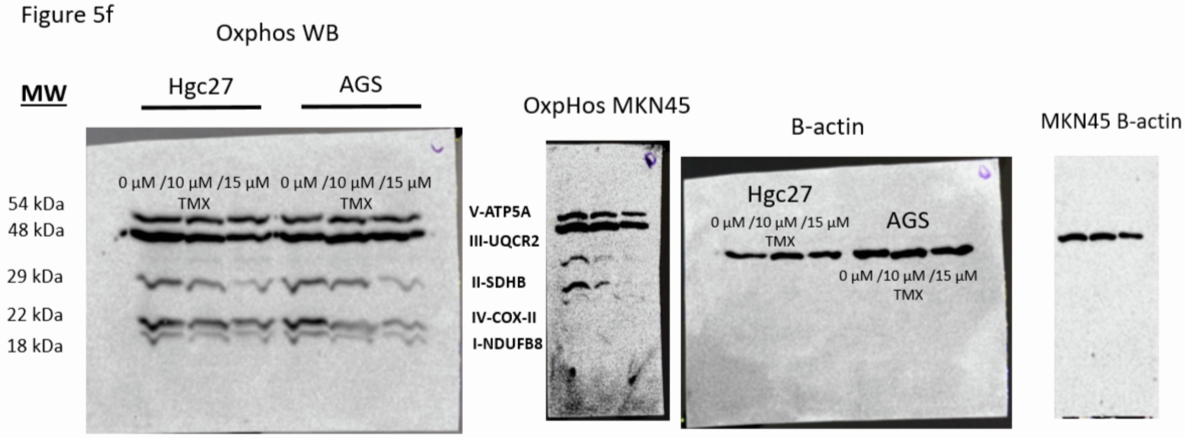

DATA S3

High Resolution immunohistochemistry images

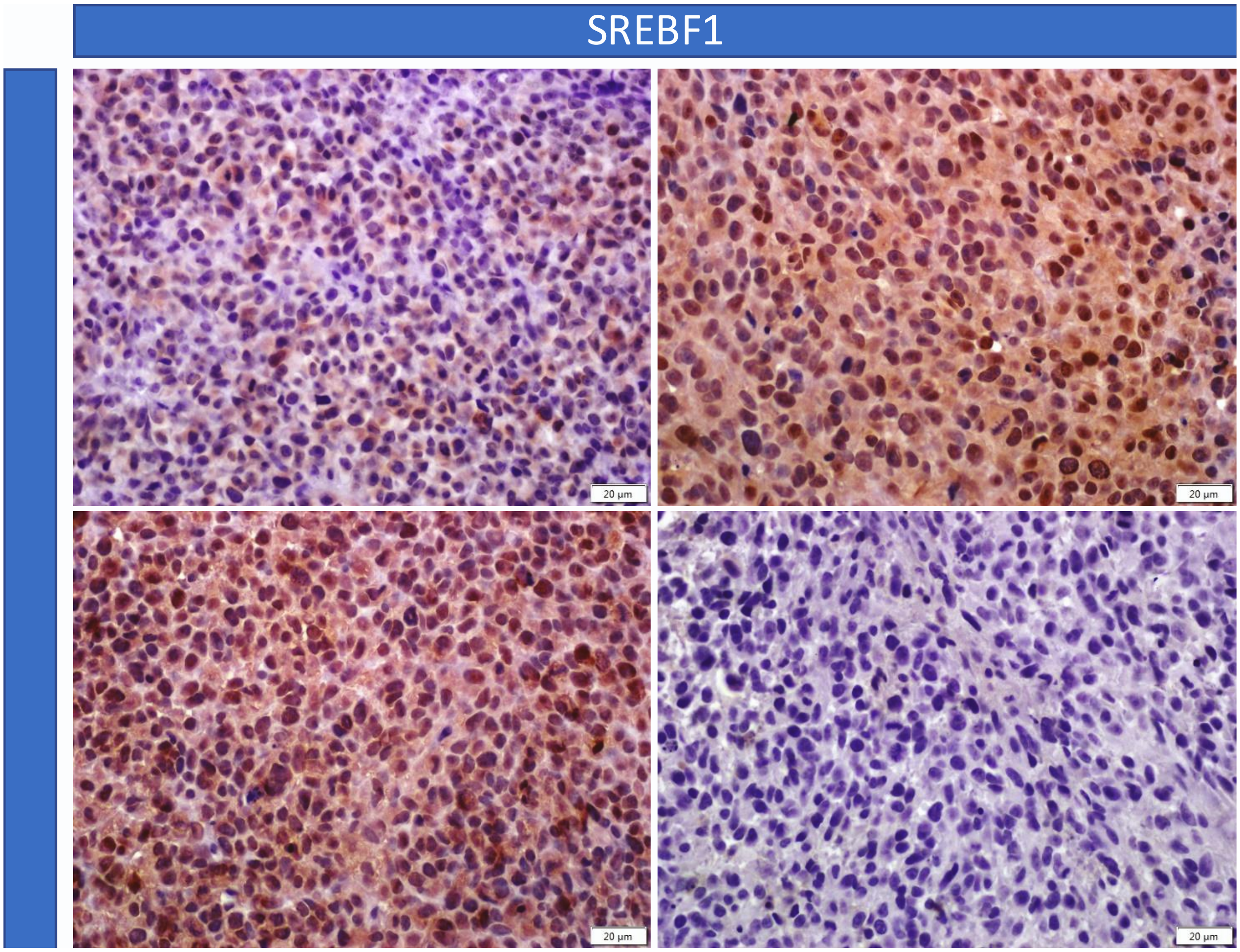

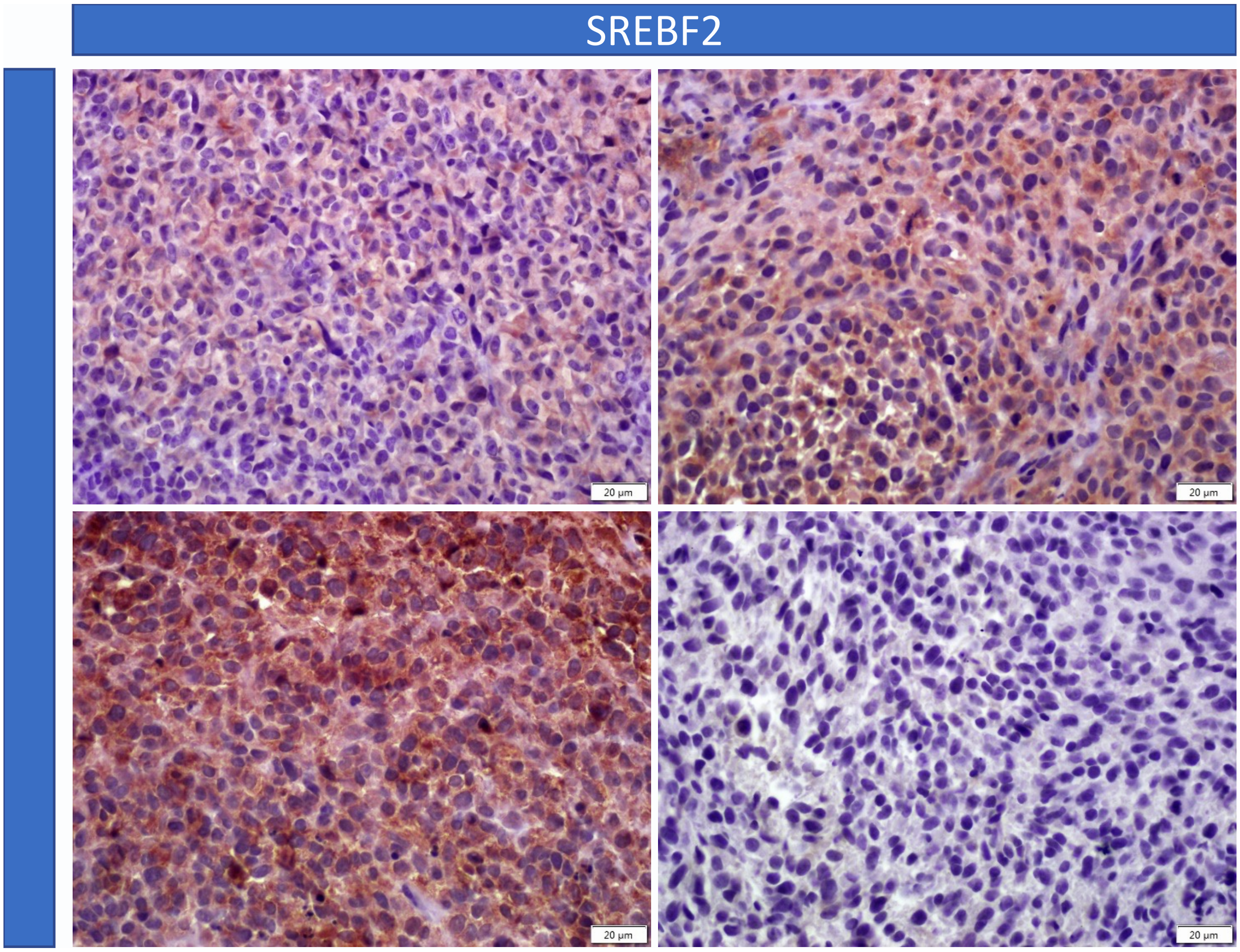
SREBF2
20 µm
20 µm
20 µm
20 µm

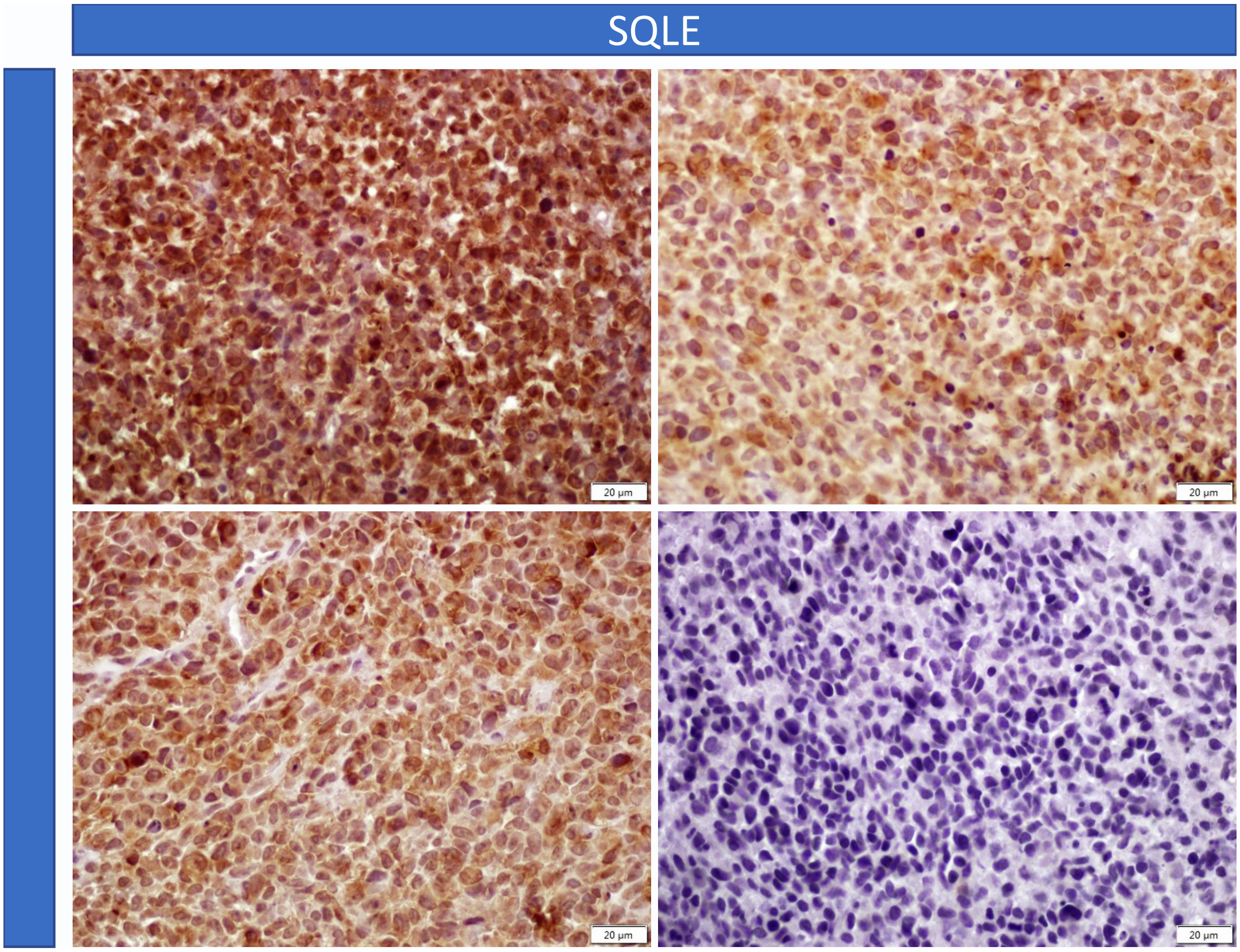
SQLE
20 µm
20 µm
20 µm
20 µm

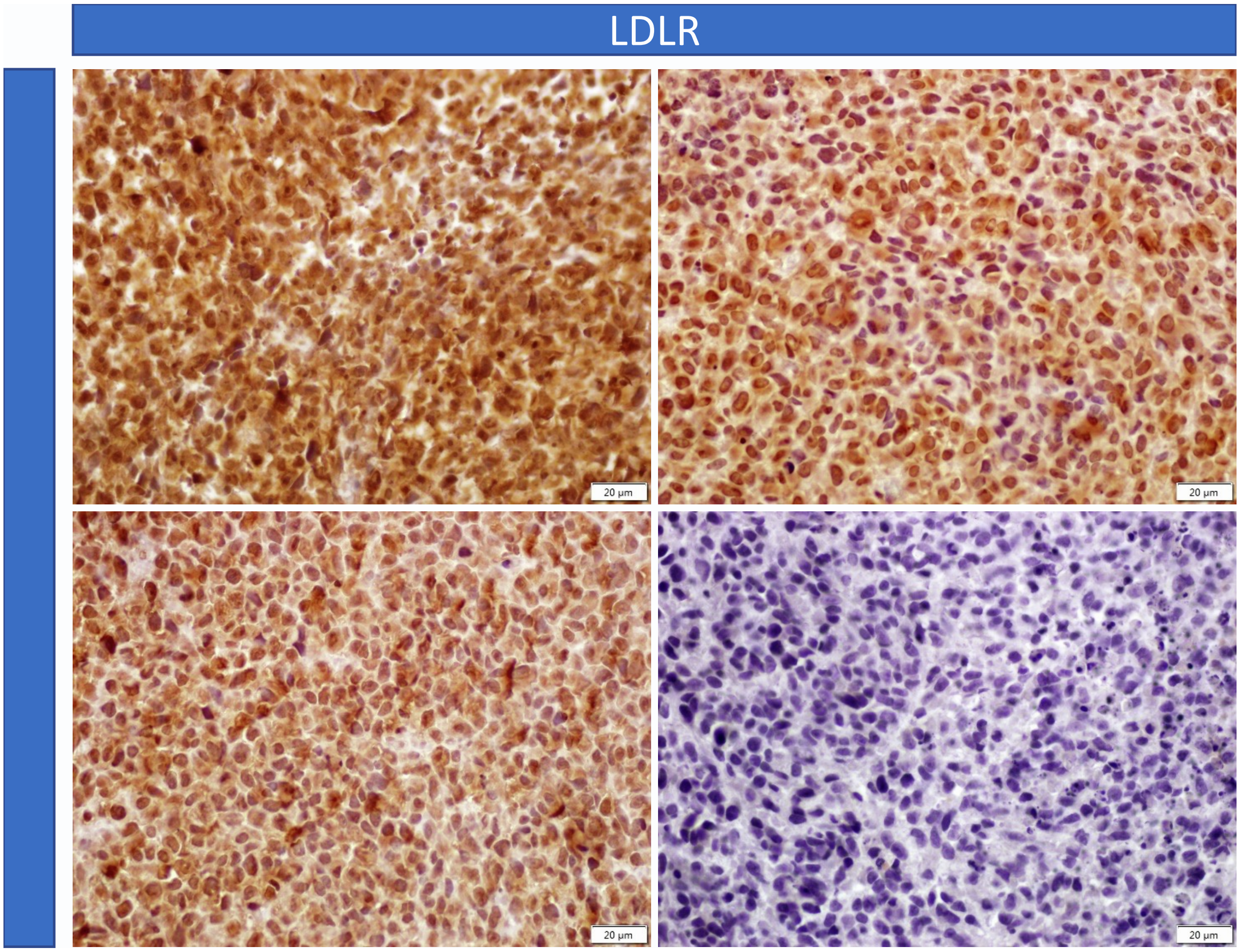
LDLR
20 µm
20 µm
20 µm
20 µm

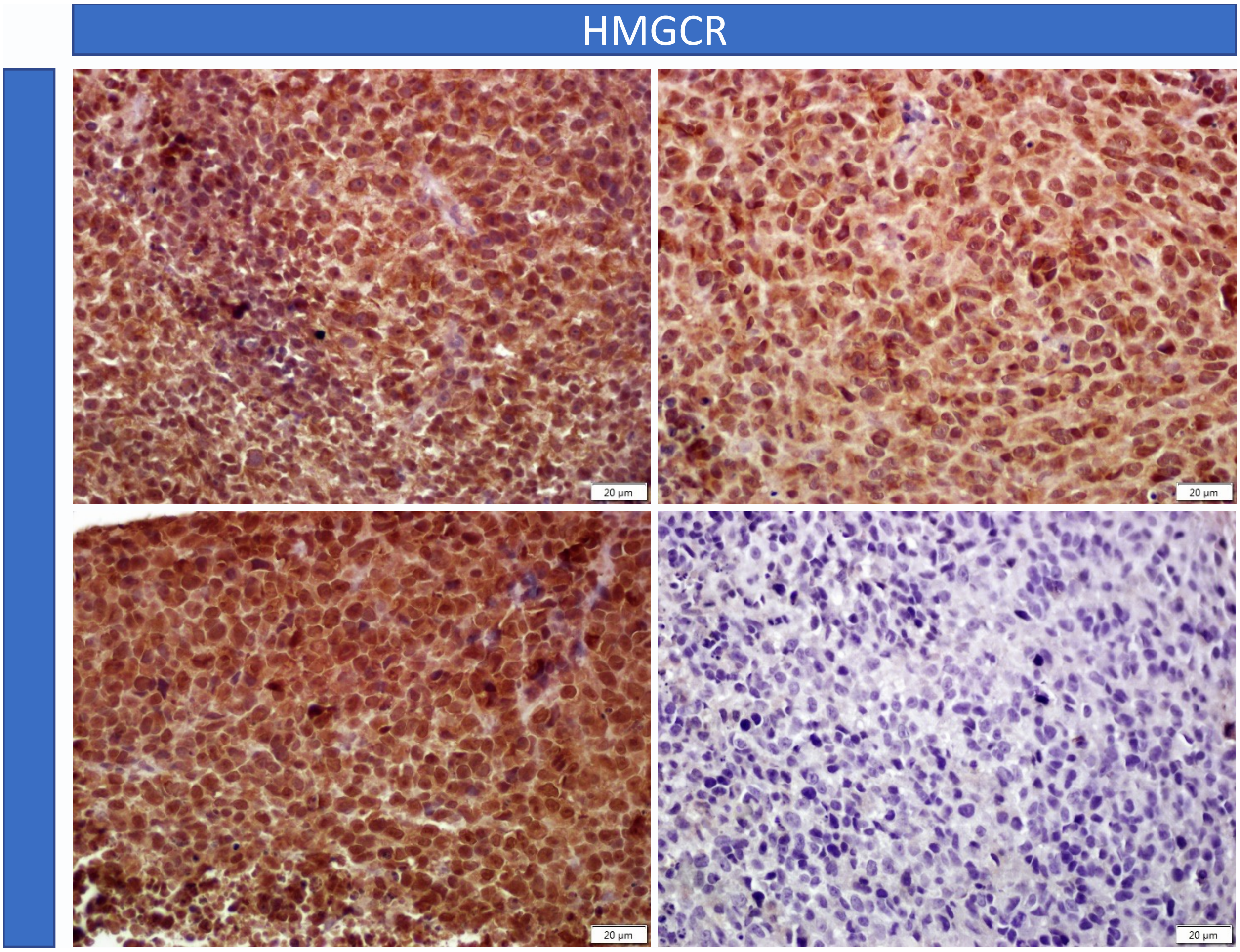
HMGCR
20 μm
20 μm
20 μm
20 μm
